# Regulation of Oncogenic Targets by Tumor-Suppressive *miR-150-3p* in Lung Squamous Cell Carcinoma

**DOI:** 10.3390/biomedicines9121883

**Published:** 2021-12-11

**Authors:** Keiko Mizuno, Kengo Tanigawa, Shunsuke Misono, Takayuki Suetsugu, Hiroki Sanada, Akifumi Uchida, Minami Kawano, Kentaro Machida, Shunichi Asai, Shogo Moriya, Hiromasa Inoue, Naohiko Seki

**Affiliations:** 1 Department of Pulmonary Medicine, Graduate School of Medical and Dental Sciences, Kagoshima University, Kagoshima 890-8520, Japan; keim@m.kufm.kagoshima-u.ac.jp (K.M.); k8802984@kadai.jp (K.T.); k8574402@kadai.jp (S.M.); taka3741@m2.kufm.kagoshima-u.ac.jp (T.S.); k8173956@kadai.jp (H.S.); akiuchi@m3.kufm.kagoshima-u.ac.jp (A.U.); k6088046@kadai.jp (M.K.); machida@m.kufm.kagoshima-u.ac.jp (K.M.); inoue@m2.kufm.kagoshima-u.ac.jp (H.I.); 2 Department of Functional Genomics, Chiba University Graduate School of Medicine, Chuo-ku, Chiba 260-8670, Japan; cada5015@chiba-u.jp; 3 Department of Biochemistry and Genetics, Chiba University Graduate School of Medicine, Chuo-ku, Chiba 260-8670, Japan; moriya.shogo@chiba-u.jp

**Keywords:** microRNA, *miR-150-3p*, passenger strand, lung squamous cell carcinoma, *HELLS*, Gene Ontology

## Abstract

Several recent studies have shown that both strands of certain miRNAs derived from miRNA duplexes are involved in cancer pathogenesis. Our own recent studies revealed that both strands of the *miR-150* duplex act as tumor-suppressive miRNAs in lung adenocarcinoma (LUAD) through the targeting of several oncogenes. The aim of the study here was to further investigate the tumor-suppressive roles of *miR-150-3p* (the passenger strand) in lung squamous cell carcinoma (LUSQ) and its control of cancer-promoting genes in LUSQ cells. The downregulation of *miR-150-3p* in LUSQ tissues was confirmed by data in The Cancer Genome Atlas (TCGA). The ectopic expression of *miR-150-3p* attenuated cancer cell aggressive features, e.g., cell cycle arrest, migration and invasive abilities. Our target search strategy successfully identified a total of 49 putative targets that were listed as subjects of *miR-150-3p* regulation in LUSQ cells. Interestingly, among these targets, 17 genes were categorized as related to the “cell cycle” based on Gene Ontology (GO) classification, namely *CENPA*, *CIT*, *CCNE1*, *CCNE2*, *TIMELESS*, *BUB1*, *MCM4*, *HELLS*, *SKA3*, *CDCA2*, *FANCD2*, *NUF2*, *E2F2*, *SUV39H2*, *CASC5*, *ZWILCH* and *CKAP2*). Moreover, we show that the expression of *HELLS* (helicase, lymphoid specific) is directly controlled by *miR-150-3p*, and its expression promotes the malignant phenotype of LUSQ cells.

## 1. Introduction

Lung cancer is the deadliest cancer in the world, and approximately 1.8 million patients died from this disease in 2018 [1,2]. Lung cancer is histologically categorized into two types: non-small cell lung cancer (NSCLC) and small cell lung cancer. Approximately 85% of all lung cancers are NSCLC, which is further classified into three subtypes: adenocarcinoma (LUAD), squamous cell carcinoma (LUSQ) and large cell carcinoma [3].

The latest genomic analyses have revealed the presence of therapeutic target genes for LUAD, e.g., *EGFR*, *MET* and *BRAF* mutations and *RET, ALK* and *ROS1* rearrangements. Treatment based on driver mutations was recently performed, resulting in the improved prognosis of patients with LUAD [4,5,6,7,8,9]. Even with intensive genomic analysis using next generation sequencing (NGS), the search for therapeutic targets for LUSQ has been difficult and is, thus, an urgent task.

MicroRNAs (miRNAs) belong to the class of noncoding RNAs and are found in all species [10,11]. They are involved in various biological processes, including fine tuning of gene expression. A single miRNA species is involved in controlling the expression of numerous genes [10,12]. Therefore, aberrant expression of a single miRNA can have a range of effects on orderly gene expression. A vast number of studies reveal that dysregulated miRNAs cause human diseases, including cancers [13,14,15,16,17]. Aberrant expression of miRNAs is deeply involved in human cancers, e.g., cancer cell progression, metastasis and drug resistance [18,19]. Therefore, the use of miRNAs as therapeutic agents (inhibitors and replacements) is very attractive. Recent developments in nanotechnology have made it possible to delivery miRNAs into diseased cells, e.g., lipid-based carriers, cationic polymer-based carriers and exosome-based carriers [20]. However, the fact that a single miRNA affects many target molecules has become a problem in clinical trial [21].

In general, the theory of miRNA biogenesis states that two types of miRNAs are derived from the pre-miRNA duplex. One is incorporated into the RNA-induced silencing complex (RISC) and controls the expression of target genes, defined as the “guide strand”. The miRNA complementary to the guide strand miRNA (called the passenger strand) is generally degraded in the cytoplasm [22].

An RNA-sequencing-based approach is suitable for creating cancer signatures based on miRNA expression. We recently established a number of miRNA expression signatures for pancreatic ductal adenocarcinoma (PDAC), head and neck squamous cell carcinoma (HNSCC), esophageal squamous cell carcinoma (ESCC) and breast cancer [23,24,25,26]. Our miRNA expression signatures revealed that both the guide and passenger strands of some miRNAs were significantly reduced in cancer tissues [27,28]. Moreover, our functional assays using ectopic expression of miRNAs showed that some miRNA passenger strands (e.g., *miR-101-5p*, *miR-143-5p* and *miR-199a-3p*) had tumor-suppressive functions via the direct control of oncogenic genes [27,29,30]. Therefore, elucidation of the passenger strands that control genes is a new challenge in understanding the molecular pathogenesis of cancer cells.

Our previous study demonstrated that *miR-150-5p* (the guide strand) acts as a tumor-suppressive miRNA in LUSQ cells. Matrix metalloproteinase 14 (*MMP14*) was found to be a direct target of *miR-150-5p*, and its overexpression promotes LUSQ cell migration and invasion [31]. Our present data indicate that *miR-150-3p* (the passenger strand) has a tumor-suppressive function, similarly to *miR-150-5p*. Interestingly, *miR-150-3p*-controlled genes were observed to be involved in cell cycle-related activities. Examining genes that are controlled by miRNAs (including passenger strand) should enhance our knowledge of the molecular pathology of LUSQ.

## 2. Materials and Methods

### 2.1. Human LUSQ Cell Lines and Cell Culture

LUSQ cell lines (EBC-1 and SK-MES-1) were obtained from the Japanese Cancer Research Resources Bank (JCRB) (Osaka, Japan) and the American Type Culture Collection (ATCC) (Manassas, VA, USA) respectively. EBC-1 cells were grown in RPMI-1640 medium (FUJIFILM Wako Pure Chemical Corporation, Osaka, Japan) supplemented with 10% fetal bovine serum (FBS). SK-MES-1 cells were grown in EMEM medium (ATCC) supplemented with 10% FBS. Both cell lines were incubated at 37 °C in a 5% CO_2_ atmosphere. Cell maintenance was performed as described in our previous publications [27,28,31,32]. 

### 2.2. RNA Extraction and Quantitative Reverse Transcription Polymerase Chain Reaction (qRT-PCR)

Isogen II (NIPPON GENE Co., Ltd., Tokyo, Japan) was used to extract total RNA from the cell lines. A NanoDrop 2000c spectrophotometer (Thermo Fisher Scientific Inc., Waltham, MA, USA) was used for assessment of total RNA quantification and quality. PrimeScript^TM^ RT Master Mix (catalog no.: RR036A, Takara Bio Inc., Shiga, Japan) and High Capacity cDNA Reverse Transcription Kit (Applied Biosystems, Foster City, CA, USA) were used to synthesize cDNA. TaqMan Real-Time PCR Assays were performed to analyze miRNA and gene expression according to our previous studies [27,28,31,32]. StepOnePlus Real-Time PCR System (Applied Biosystems) was used to run qRT-PCR reactions. The data were assessed in triplicate wells. Details of TaqMan probes and primers are shown in Table S1. 

### 2.3. Transfection of miRNAs, siRNAs and Plasmid Vectors into LUSQ Cells

The miRNAs and siRNAs were transfected with Opti-MEM (Gibco, Carlsbad, CA, USA) and Lipofectamine^TM^ RNAiMax Transfection Reagent (Invitrogen, Carlsbad, CA, USA) into LUSQ cell lines. Plasmid vectors were transfected into cells using Lipofectamine^TM^ 2000 Transfection Reagent (Invitrogen). The procedures for miRNA, siRNA, and plasmid vector transfection into LUSQ cells were as previously described [27,28,31,32]. The miRNAs and siRNAs were transfected for 96 h in XTT assays and 72 h in the other experiments. The reagents using in this study are listed in Table S1. 

### 2.4. Functional Assays for LUSQ Cells (Cell Proliferation, Migration, Invasion and Cell Cycle)

XTT assays using a Cell Proliferation Kit (Biological Industries, Beit-Haemek, Israel) were performed to assess cell proliferation. Cell migration was measured using wound healing assays. Cells were plated in six-well plates, and the cell monolayer was scraped using PIPETMAN DIAMOND Tips D200 (Gilson Incorporated Global Headquarters, Middleton, WI, USA) after transfection. The gap lengths 0 and 24 h after wounding were measured on photomicrographs. Cell invasion assessment was conducted using Corning BioCoat^TM^ Matrigel Invasion Chambers (CORNING, Bedford, MA, USA). Flow cytometry (BD FACSCelesta^TM^ Flow Cytometer, BD Biosciences, Franklin Lakes, NJ, USA) using BD Cycletest^TM^ Plus DNA Reagent Kits (BD Biosciences) was performed to evaluate the cell cycle. The results were analyzed using BD FACSDiva software (version 8.0.1.1, BD Biosciences). Cell proliferation, migration, invasion and cell cycle assays were performed as described in our previous publications [27,31]. All experiments were performed in triplicate. 

### 2.5. Identification of Oncogenic Targets by miR-150-3p Regulation in LUSQ Cells

The TargetScanHuman database (http://www.targetscan.org/vert_72/, accessed on 14 August 2019) was used to predict miRNA-binding sites. The expression profiles of *miR-150-3p*-transfected EBC-1 and SK-MES-1 cells were created, and expression data were deposited in the Gene Expression Omnibus (GEO) database (accession number: GSE163187). 

### 2.6. In Silico Analysis of miRNA and Gene Expression Levels 

The Cancer Genome Atlas (TCGA) datasets (https://www.cancer.gov/tcga) were used to assess the clinical significance of miRNAs and their target genes in lung squamous cell carcinoma. The gene expression levels and clinical data were obtained from cBioPortal (https://www.cbioportal.org/) and OncoLnc (http://www.oncolnc.org/) (data downloaded on 28 December 2019). 

### 2.7. Plasmid Construction and Dual-Luciferase Reporter Assays

We prepared psiCHECK-2 plasmid vectors (catalog no.: C8021, Promega, Madison, WI, USA) which have a wild-type or a deletion-type of the *miR-150-3p*-binding site in the coding region of *HELLS*. *miR-150-3p* and the plasmid vectors were transfected into lung cancer cells for 36 hours. The Dual-Luciferase Reporter Assay System (Promega) was used to assess firefly and *Renilla* luciferase activities. Luciferase reporter assays were evaluated in triplicate wells. The dual-luciferase reporter assay procedure was described in our previous studies [27,31,32]. 

### 2.8. Western Blotting

The method for western blotting has been described in past reports [27,31,32]. In brief, RIPA Lysis Buffer System (catalog no.: sc-24948, Santa Cruz Biotechnology Inc., Dallas, TX, USA) was used to lyse cells. Protein concentrations were measured using the Pierce^TM^ BCA Protein Assay Kit (Thermo Fisher Scientific, Rockford, IL, USA). Protein (20 μg) was injected into each well of SuperSep^TM^ Ace (6%, 13 well) (FUJIFILM Wako Pure Chemical Corporation, Osaka, Japan) and electrophoresis was performed. Precision Plus Protein^TM^ Dual Color Standards were used as molecular weight markers. Proteins were transferred onto polyvinylidene fluoride membranes (catalog no.: PPVH00010, Merck KgaA, Darmstadt, Germany). The membranes were incubated with blocking buffer (3% bovine serum albumin or 5% fat-free milk). After blocking, they were sequentially incubated, first with the primary antibody and subsequently in the blocking buffer containing the secondary antibody. Precision Protein^TM^ Strep Tactin-HRP Conjugate (catalog no.: #1610380, Bio-Rad Laboratories, Inc., Hercules, CA, USA) was used for chemiluminescent detection. The antibodies used in this study are shown in Table S1. Amersham ECL Prime Western Blotting Detection Reagent (Cytiva; Marlborough, MA, USA) was used to develop the signal. Visualization of the western blot was by chemiluminescence using FluorChem FC2 (Cell Biosciences, Santa Clara, CA, USA). 

### 2.9. Immunohistochemical Staining

The procedure for immunohistochemical staining (IHC) was as previously described [27,28,31,32]. The tissue microarray slide (catalog no.: LC811a, US Biomax, Inc. Derwood, MD, USA) was de-waxed with xylene and ethanol. The slide was heated in 10× citrate buffer for heat epitope recovery, pH 6.0 (Diagnostic BioSystems, Pleasanton, CA, USA) for activation. After that, it was immersed in 0.3% H_2_O_2_ to block peroxidase activity. The antigen–antibody complex was detected by VECTASTAIN Universal Elite ABC Kit (Vector Laboratories, Burlingame, CA, USA). Primary antibody was diluted with Dako Real antibody diluent (Agilent, Santa Clara, CA, USA). Dako REAL^TM^ EnVision^TM^ Detection System Peroxidase/DAB+, rabbit/mouse (Agilent) was used in a chromogenic method. The primary antibody is described in Table S1. Two pathologists independently evaluated the immunostaining of lung cancer tissue. The IHC intensity was quantified on the basis of positive control. 

### 2.10. Statistical Analysis

Statistical analyses were performed using GraphPad Prism 8 (GraphPad Software, La Jolla, CA, USA) and JMP Pro 14 (SAS Institute Inc., Cary, NC, USA). Multiple group comparison was achieved using one-way analysis of variance (ANOVA) and Tukey’s post hoc test. The significance of differences between two groups was assessed by Mann–Whitney U tests.

## 3. Results

### 3.1. Expression Levels of miR-150-5p and miR-150-3p in Clinical LUSQ Tissues

**Figure 1:**
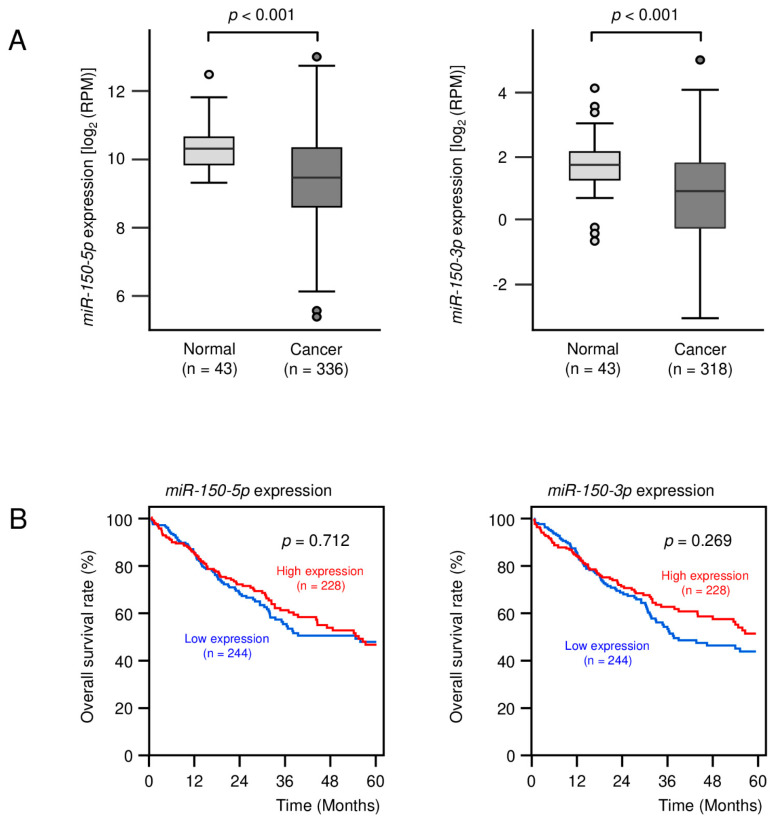
Expression levels of *miR-150-5p* and *-3p* and prognosis due to the difference in expression levels in LUSQ. (**A**) Expression levels of *miR-150-5p* and *miR-150-3p* in normal and cancer specimens were assessed using The Cancer Genome Atlas (TCGA). (**B**) Relationships between the expression levels of *miR-150-5p* and *miR-150-3p* and Kaplan–Meier curves of 5-year overall survival in LUSQ patients were evaluated using TCGA database.

The expression levels of *miR-150-5p* and *miR-150-3p* were evaluated by TCGA database analysis. The expression levels of both *miR-150-5p* and *miR-150-3p* were significantly downregulated in LUSQ tissues compared with normal tissues (both, *p* < 0.001; Figure 1A). 

Cohort analyses using the TCGA-LUSQ datasets showed that the expression of *miR-150-5p* and *miR-150-3p* did not affect the 5-year survival rate of the patients (Figure 1B).

### 3.2. Tumor-Suppressive Roles of miR-150-5p and miR-150-3p in LUSQ Cells

**Figure 2:**
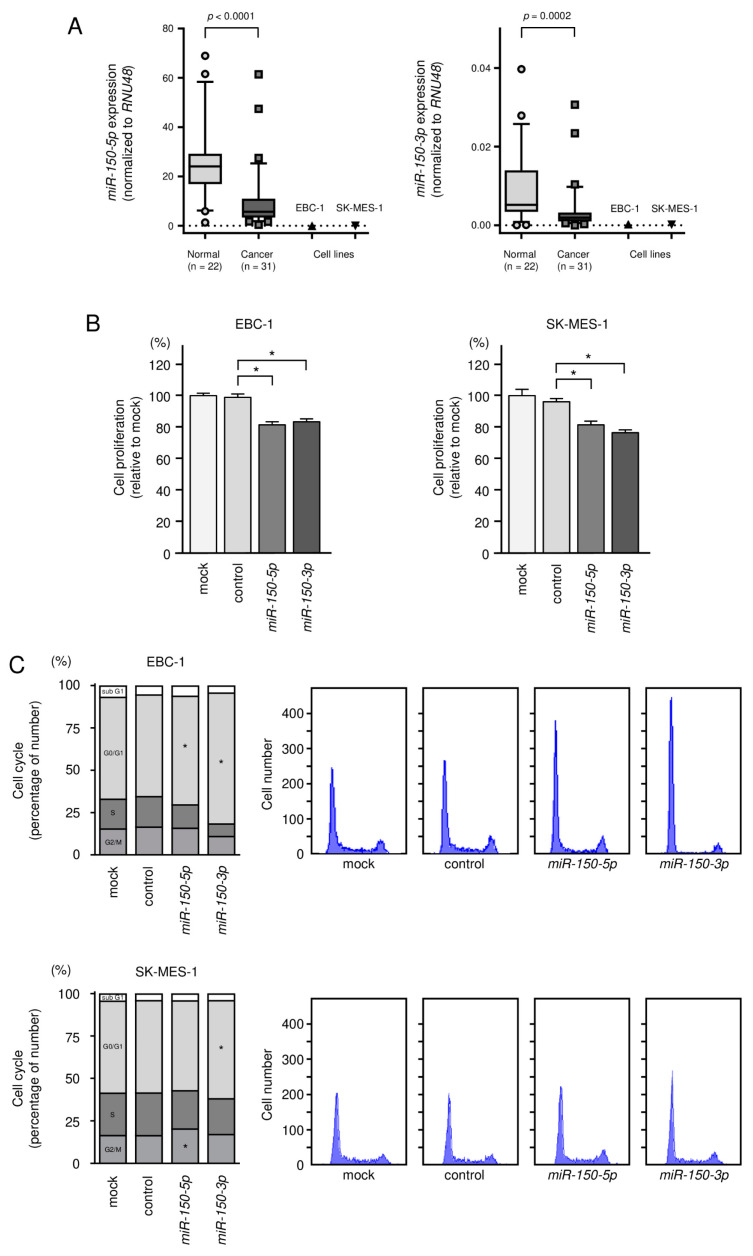
Expression levels of *miR-150-5p* and *miR-150-3p* in LUSQ clinical specimens and LUSQ cell lines and effects of ectopic expression of *miR-150-5p* and *miR-150-3p* in LUSQ cells. (**A**) Expression levels of *miR-150-5p* and *miR-150-3p* were assessed in clinical specimens and cell lines (EBC-1 and SK-MES-1). Data were normalized to *RNU48*. (**B**) XTT assays were performed after transfection of *miR-150-5p* or *miR-150-3p* to assess cell proliferation. (**C**) Cell cycle status after transfection of *miR-150-5p* or *miR-150-3p* was tested by flow cytometry. * *p* ≤ 0.0001.

For the validity of the gain-of-function assays, we examined the expression of *miR-150-5p* and *miR-150-3p* in the LUSQ cell lines EBC-1 and SK-MES-1. It was detected that the expression of *miR-150-5p* and *miR-150-3p* in the cell line was significantly lower than that of normal lung tissues (Figure 2A). Clinical information for the normal lung tissue is shown in Tables S2 and S3.

**Figure 3:**
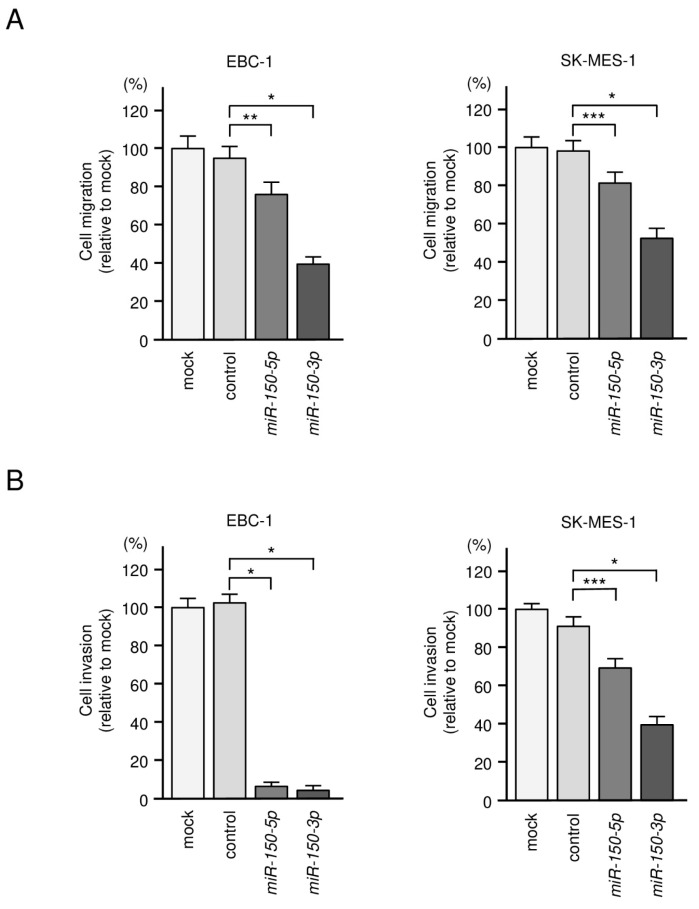
Effects of ectopic expression of *miR-150-5p* and *miR-150-3p* in LUSQ cells. (**A**) Wound healing assays were performed after transfection with *miR-150-5p* or *miR-150-3p* to measure cell migration. (**B**) Matrigel invasion assay was performed after transfection with *miR-150-5p* or *miR-150-3p* to determine cell invasion. * *p* < 0.0001, ** *p* < 0.001, *** *p* < 0.05.

We assessed cell proliferation, cell cycle, migration and invasion based on transient transfection assays. Ectopic expression of the two miRNAs inhibited cancer cell proliferation in EBC-1 and SK-MES-1 cells (Figure 2B). Cell cycle assays showed that expression of *miR-150-3p* induced G0/G1 arrest in EBC-1 and SK-MES-1 cells (Figure 2C). Moreover, expression of *miR-150-5p* and *miR-150-3p* significantly blocked the migration and invasive abilities of LUSQ cells (Figure 3A,B and Figure S1). 

Our data suggest that both *miR-150-5p* and *miR-150-3p* may have important role as tumor suppressors through downregulation of target oncogenic genes. We investigated the synergistic effect when both miRNAs were simultaneously transfected into cancer cells. However, we found no synergistic effect on cell viability inhibition based on *miR-150-5p* and *miR-150-3p* co-transfection (Figure S2). In the future, it will be necessary to conduct the analysis using an expression system that simultaneously expresses both miRNAs in cancer cells.

These data indicate that both strands of pre-*miR-150* act as tumor-suppressive miRNAs in LUSQ cells. In contrast to *miR-150-5p* (the guide strand), there are not many reports of oncogenic genes in LUSQ cells that are controlled by *miR-150-3p* (the passenger strand). Therefore, we searched for oncogenic targets controlled by *miR-150-3p* in LUSQ cells. 

### 3.3. Identification of Oncogenic Targets Regulated by miR-150-3p in LUSQ Cells

**Figure 4:**
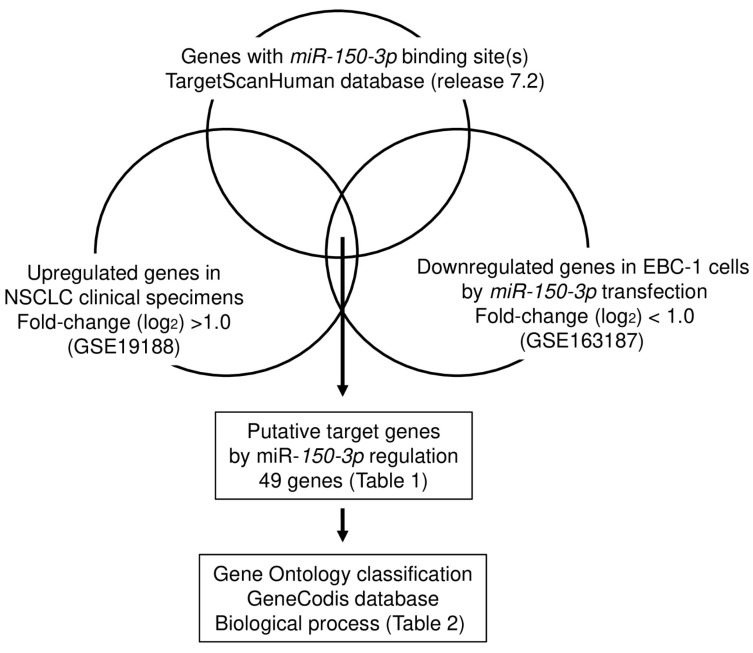
Flowchart for searching for oncogenic targets subject to *miR-150-3p* regulation in LUSQ cells. To identify genes controlled by *miR-150-3p* in LUSQ cells, we used the TargetScanHuman database and two gene expression profile datasets (GES19188 and GSE163187). A total of 49 genes were identified as possibly controlled by *miR-150-3p*.

In searching for oncogenic genes controlled by *miR-150-3p* in LUSQ cells, we made the following hypotheses: (1) target genes of *miR-150-3p* have one or more binding site(s), (2) target genes are upregulated in LUSQ tissues and (3) target genes will be downregulated following *miR-150-3p* transfection of LUSQ cells. We searched for genes that satisfied the three conditions by combining in silico analysis (TargetScanHuman database and GSE19188) and our gene expression data (GSE163187). A flowchart of the strategy used to narrow down the list of candidate genes is shown in Figure 4. 

A total of 49 putative targets of *miR-150-3p* in LUSQ were identified (Table 1). These genes were additionally categorized according to Gene Ontology classification. Interestingly, the target molecules were significantly enriched for genes related to the “cell cycle” and “cell division” categories (Table 2). 

**Figure 5:**
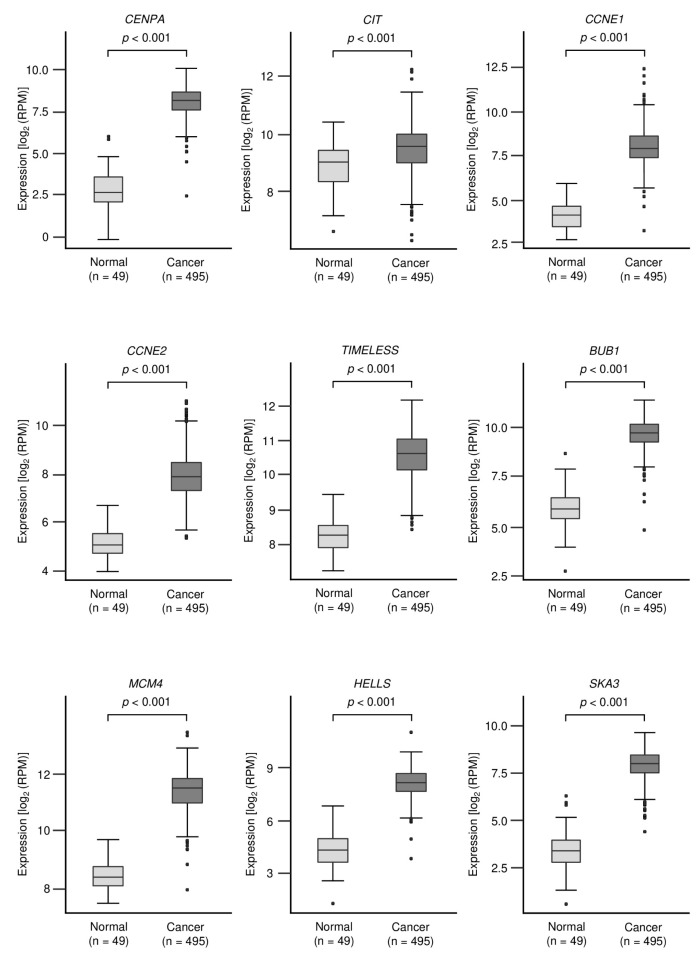

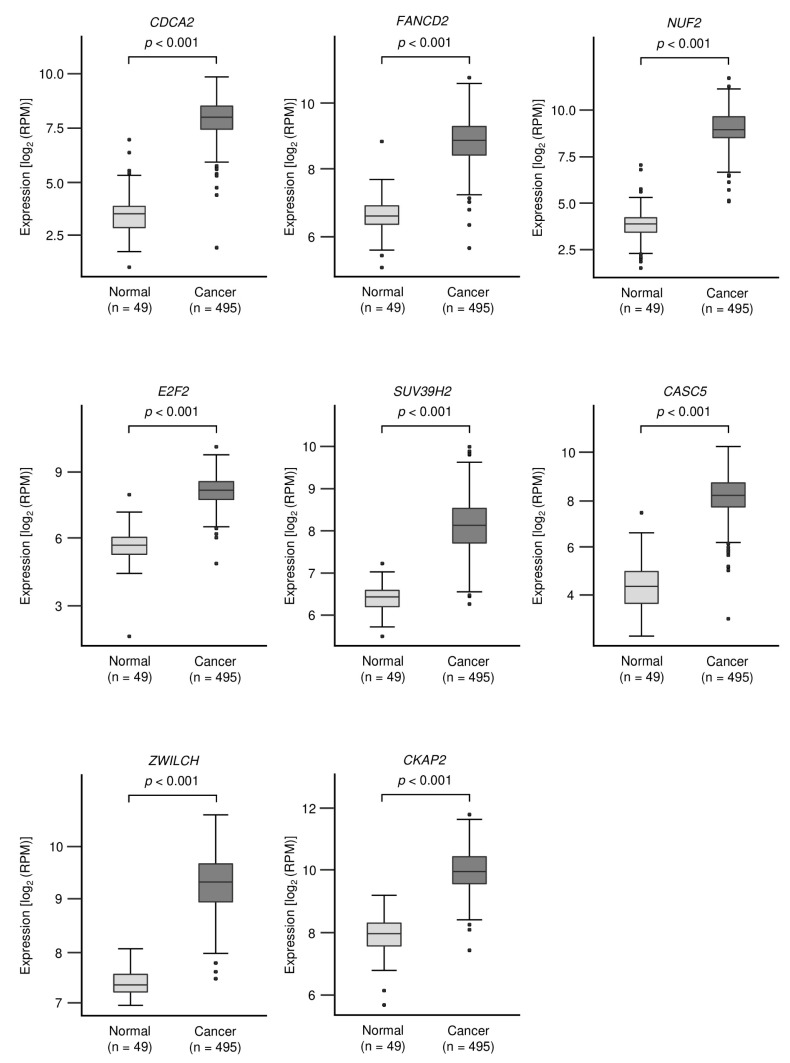
Expression levels of *miR-150-3p* target genes in LUSQ tissues. Analysis of the expression levels of *miR-150-3p* target genes involved in the “cell cycle” and “cell division” pathways. A total of 17 genes were evaluated in LUSQ clinical specimens using TCGA database.

The expression levels of genes involved in the “cell cycle” and “cell division” were evaluated by TCGA Database analysis. All genes were found to be significantly upregulated in LUSQ tissues (*p* < 0.001, Figure 5).

### 3.4. Direct Regulation of HELLS by miR-150-3p in LUSQ Cells

In this study, we focused on *HELLS* for the following reasons: (1) TargetScanHuman database analysis showed that a *miR-150-3p* binding site was present within the coding region of the *HELLS* gene. Generally, miRNA binding sites are located in the 3′UTR of the genes. (2) OncoLnc database analysis showed that the prognosis of patients with LUAD and patients with LUSQ differs depending on *HELLS* expression. (3) The functional analysis of *HELLS* has not been investigated in LUSQ cells.

**Figure 6:**
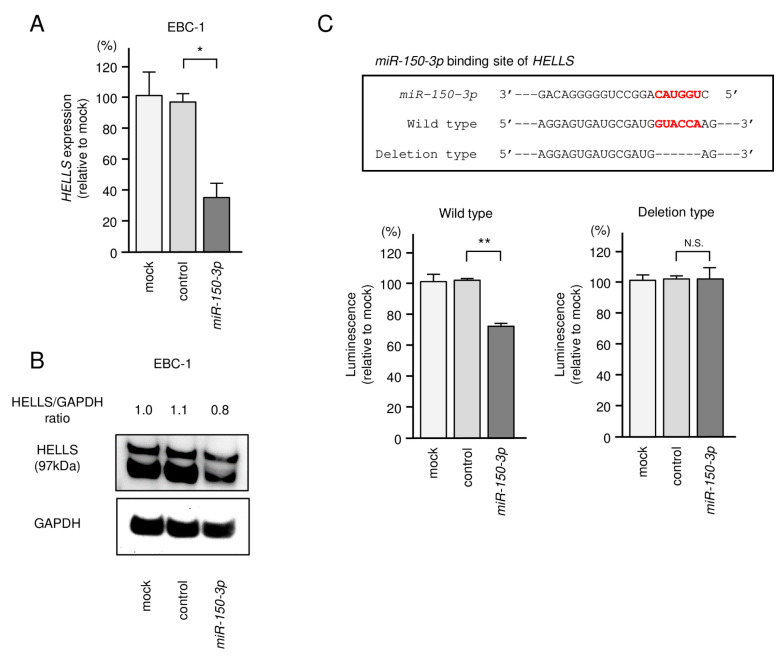
Direct regulation of HELLS by *miR-150-3p* in LUSQ cells. (**A**) qRT-PCR was performed after transfection of *miR-150-3p* to validate the mRNA expression of *HELLS*. *GUSB* was used as an internal control. (**B**) Western blotting of HELLS and GAPDH was performed after transfection of *miR-150-3p*. GAPDH was used as a loading control. ((**C**), upper) The *miR-150-3p* binding site of *HELLS* was predicted using TargetScanHuman database ver.7.2. ((**C**), lower) The luciferase reporter assay confirms the presence of an *miR-150-3p* binding site in *HELLS*. Normalized data were calculated as *Renilla*/firefly luciferase activity ratios. * *p* < 0.001, ** *p* < 0.01, N.S.: not significant.

In EBC-1 cells transfected with *miR-150-3p*, both *HELLS* mRNA and HELLS protein levels were significantly downregulated (Figure 6A,B). Full-sized images of western blot data are shown in Figure S3.

Analysis of the NCBI nucleotide and TargetScanHuman databases revealed that the *miR-150-3p* binding site (CAUGGU) was located within the coding region of *HELLS* gene (Figure S4). The presence of the *miR-150-3p* binding site within the coding region in the LUSQ and LUAD cell lines (EBC-1, SK-MES-1, A549 and H1299) was confirmed through sequencing (Figure S5).

Next, we performed a luciferase reporter assay to determine whether *miR-150-3p* directly binds to *HELLS*. Luciferase activity was significantly reduced in cells transfected with the wild-type vector (with *miR-150-3p* binding site) but not in cells transfected with the deletion construct (without *miR-150-3p* binding site) (Figure 6C). Our present data demonstrate that *miR-150-3p* directly bound to its target sequence *HELLS* and negatively controlled its expression in LUSQ cells. The sequence used for luciferase reporter assays is shown in Figure S5.

### 3.5. Effects of Knockdown of HELLS in LUSQ Cells

**Figure 7:**
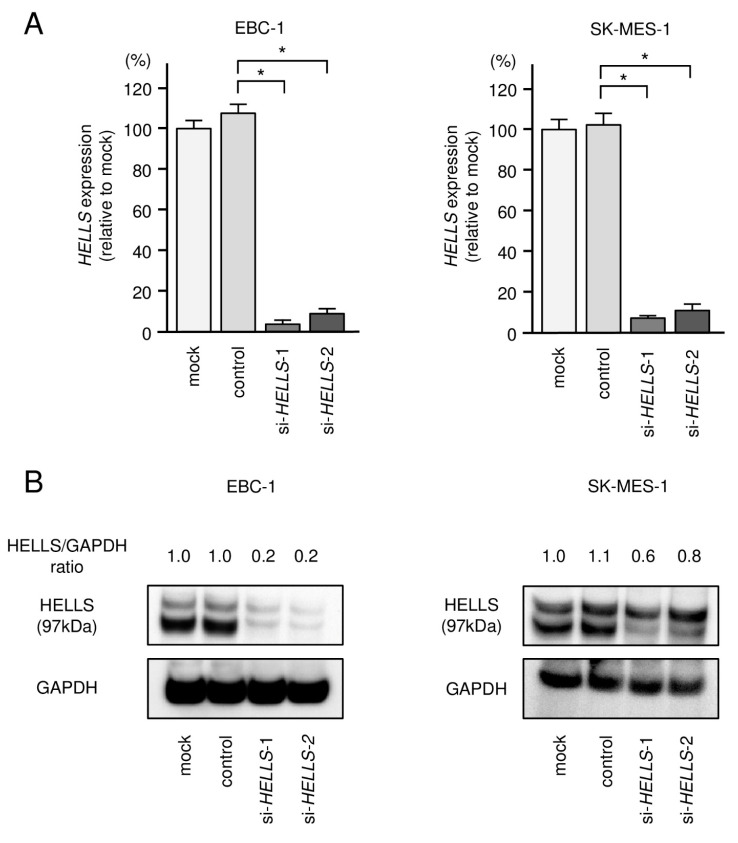
(**A**) qRT-PCR was performed to assess the expression of *HELLS* after transfection with si-*HELLS*. *GUSB* was used as the internal control. (**B**) Western blotting was carried out using anti-HELLS antibody after transfection with si-*HELLS*. GAPDH was used as the loading control. * *p* < 0.0001.

To investigate the oncogenic roles of *HELLS* in LUSQ cells, we used knockdown assays based on two types of small interfering RNAs (siRNAs) targeting *HELLS*. First, to validate the knockdown efficiencies of siRNAs in LUSQ cells, the mRNA and protein levels of HELLS were shown to be successfully downregulated by either siRNAs in EBC-1 and SK-MES-1 cells (Figure 7A,B). Full-sized images of western blot data are shown in Figure S6.

**Figure 8:**
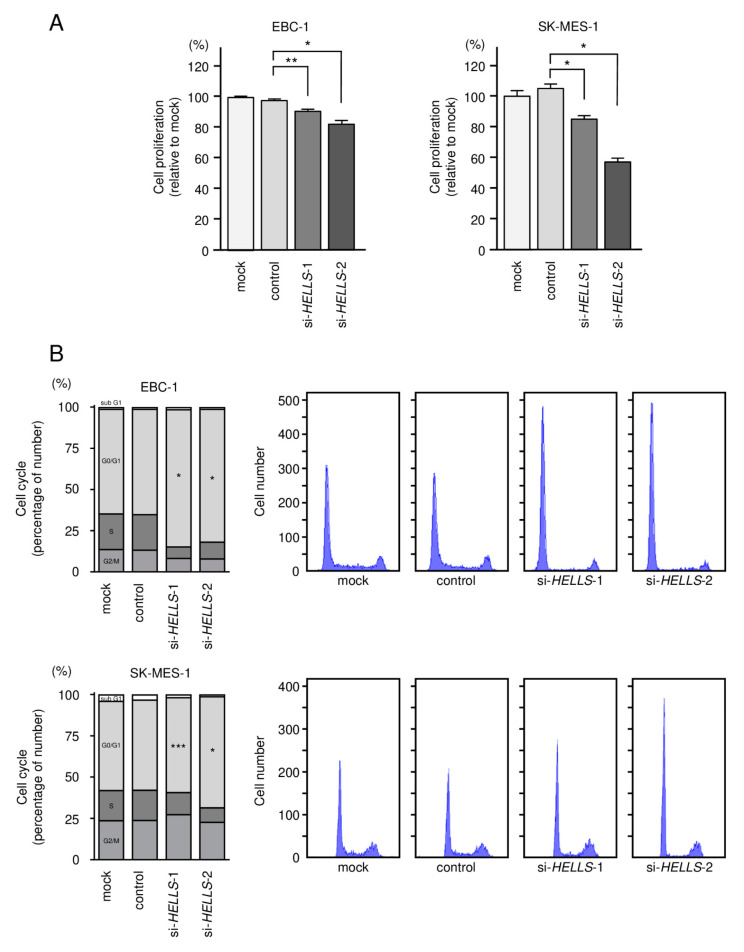
Effects of *HELLS* knockdown in LUSQ cells. (**A**) Cell proliferation was assessed using the XTT assay. (**B**) The cell cycle was analyzed by flow cytometry. G0/G1 arrest was observed after si-*HELLS* transfection. * *p* < 0.0001, ** *p* = 0.0002, *** *p* < 0.01.

**Figure 9:**
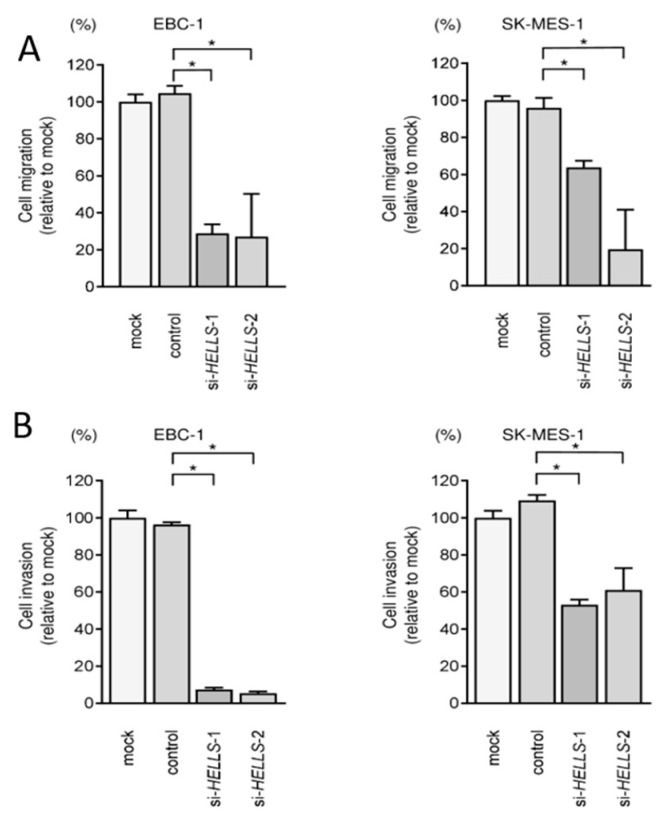
Evaluation of migration and invasion after knockdown of *HELLS* in LUSQ cells. (**A**) Wound healing assays were performed after transfection with si-*HELLS* to measure cell migration. (**B**) Matrigel invasion assays were performed after transfection with si-*HELLS* to determine cell invasion. * *p* < 0.0001.

Transient transfection of the two siRNAs into EBC-1 and SK-MES-1 cells resulted in the inhibition of cancer cell proliferation (Figure 8A). Cell cycle assays showed that expression of the two miRNAs induced G0/G1 arrest in EBC-1 and SK-MES-1 cells (Figure 8B). These results indicate that the expression of *HELLS* enhances LUSQ cell proliferation, and therefore, acts as an oncogene. Knockdown of *HELLS* in LUSQ cells also blocked migration and invasion (Figure 9). The images of migration and invasion assays are shown in Figure S7.

### 3.6. Overexpression of HELLS in LUSQ Clinical Specimens

**Figure 10:**
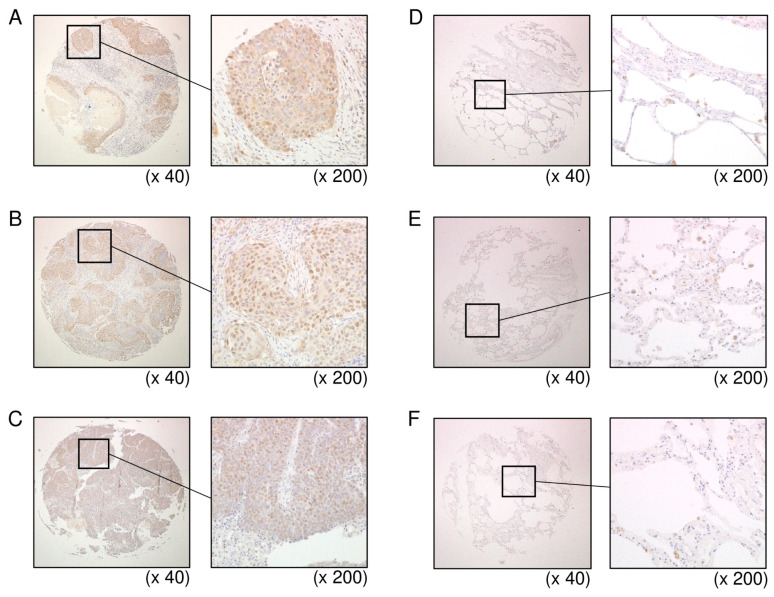
Expression of HELLS in LUSQ clinical specimens. (**A**–**C**) Overexpression of HELLS in the nuclei was observed in LUSQ tissues through immunostaining. (**D**–**F**) Weakly staining of HELLS in normal lung tissue.

Expression of HELLS protein was investigated using LUSQ tissues. It was found that HELLS was overexpressed in cancer lesions of LUSQ tissue (Figure 10A–C). In contrast to cancer lesions, HELLS expression was weak in normal lung tissues (Figure 10D–F). Table S4 provides information on the clinical specimens used for immunostaining.

Furthermore, we examined the relationship between the clinical outcome of lung cancer and *HELLS* expression using TCGA data (TCGA-LUSQ and TCGA-LUAD). In LUAD, patients with high *HELLS* expression had a significantly worse prognosis compared to those with low expression. Notably, in LUSQ, patients with low *HELLS* expression had a significantly worse prognosis compared with those with high expression (Figure S8). Forest plot presenting the results of a multivariate Cox regression analysis of the prognostic value of HELLS identified in an LUSQ and LUAD dataset from TCGA. In Figure S8, the expression level of *HELLS* was determined to be independent prognostic factors in terms of the 5-year overall survival rate after adjustments for age, sex, disease stage and pathological T and N stage (*p* < 0.05). Therefore, the expression of *HELLS* predicts opposite outcomes in patients with LUAD and LUSQ when used as a prognostic marker. At present, there is no clear explanation for this phenomenon.

## 4. Discussion

The analysis of miRNA expression signatures based on RNA sequencing revealed that some passenger strands of miRNAs (e.g., *miR-99a-3p*, *miR-101-5p*, *miR-143-5p* and *miR-145-3p*) are significantly downregulated in several types of cancer tissues and that they act as tumor suppressors in different types of cancer [26,28,29,33]. Recently, a large number of in silico analyses revealed that in some miRNAs, both strands (5p and 3p) coordinately modulate oncogenic pathways, e.g., *miR-28*, *miR-30a*, *miR-139*, *miR-143* and *miR-145* [27,28,34,35,36]. These findings show that several miRNA passenger strands are deeply associated with the molecular pathogenesis of human cancers. Simultaneous investigation of 5p and 3p strands of miRNA duplexes will elucidate novel molecular mechanism in human cancer cells.

In the last 10 years, several studies have revealed that aberrant expression of *miR-150-5p* (the guide strand) significantly contributes to human oncogenesis [31,37,38,39]. Initially, *miR-150-5p* was considered to be a hematopoietic-specific miRNA, and associations with hematological malignancies were investigated [40]. Previous studies in solid cancers showed that *miR-150-5p* behaves differently (as an oncogene or as a tumor suppressor) depending on the type of cancer [31,41]. In NSCLC, it was shown that *miR-150-5p* is overexpressed in NSCLC tissues, where it negatively regulates the tumor-suppressor TP53 [41]. Another study showed that *miR-150-5p* was present in NSCLC tissues and that its overexpression promotes cellular proliferation and migration [42]. In contrast to the oncogenic function of *miR-150-5p* in NSCLC cells, *miR-150-5p* was significantly downregulated in cancer stem cells in NSCLC [39], and the expression of *miR-150-5p* was demonstrated to inhibit the HMGA2 and β-catenin signaling pathways [39]. Our previous study supports the function of *miR-150-5p* in acting as a tumor-suppressive miRNA in LUSQ cells [31].

Few studies have simultaneously analyzed both the 5p and 3p strands of *miR-150* because passenger strands have not received much attention to date. Previous reports have demonstrated that the 5p and 3p strands of the *miR-150* duplex have tumor suppressive functions in prostate cancer, ESCC and HNSCC [37,38]. In this study, we demonstrated that ectopic expression of *miR-150-3p* (the passenger strand) attenuated the malignant phenotypes of LUSQ cells, suggesting that it acts as a tumor-suppressive miRNA. The results from both our present investigation plus our previous study show that both strands of the *miR-150* duplex act as tumor-suppressive miRNAs in LUSQ cells.

We explored the molecular networks that were controlled by *miR-150-3p* in LUSQ cells. A total of 49 genes were identified as putative targets of *miR-150-3p* regulation in LUSQ cells. Among them, 17 genes were classified as related to the “cell cycle”. For example, aberrant expression of MCM4 was detected in LUAD tissues and *MCM4* knockdown suppressed cell growth, migration and invasion of LUAD cells [27]. CENPA downregulation was found to induce G0/G1 cell cycle arrest and apoptosis. Downregulation of CENPA inhibited migration and invasion in vitro and suppressed the growth of xenografted A549 cells [43]. SKA3 induces the expression of matrix metalloproteinase (MMP)-2, -7 and -9. It also binds and activates epidermal growth factor receptor (EGFR). These processes activate the PI3K–AKT pathway and promote lung adenocarcinoma metastasis [44]. E2F transcription factors regulate cell cycle, apoptosis and DNA damage responses. E2F2 knockdown suppresses cell viability and colony formation in non-small cell lung cancer [45].

In this study, we focused on *HELLS* (helicase, lymphoid-specific), because its expression level was reduced by ectopic expression of *miR-150-3p* in LUSQ cells. Notably, *miR-150-3p* binding site was detected in coding region of *HELLS* gene. It is established concept of miRNA biogenesis that miRNAs are binding 3′-untranslated regions of their target genes, and induce mRNAs degradation [46,47]. In contrast to this theory, the molecular mechanisms by which miRNAs bind to coding regions, and regulate gene expression, is under discussion [46,47]. Several studies have indicated that miRNAs can bind to coding region of mRNAs and control of their expressions [46,47]. It has suggested that the molecular mechanisms of miRNAs expression regulation were differs depending on the site to which miRNAs bound. Previous reports suggested that when the miRNAs binding site are coding region, the major regulatory effect of miRNAs is translational inhibition [46,47]. In this study, we provided the possibility that *miR-150-3p* can bind coding region of *HELLS*, and reduced expression of *HELLS* in LUSQ cells. Additional analysis is needed on the regulatory mechanisms of gene expression for *miR-150-3p* that bind to the coding region of the genes. Other miRNAs and lncRNAs analyses involved in epigenetic regulation of *HELLS* are also required.

In this study, we focused on *HELLS* (helicase, lymphoid specific), which belongs to the SNF2 family of chromatin-remodeling ATPases [48]. HELLS is recruited to specific DNA sites to control the transcription of targeted genes [49]. Importantly, *HELLS* expression is directly controlled by the E2F1 transcription factor, and HELLS acts as an E2F3 transcriptional co-activator, resulting in the expression of cell cycle-related genes [50]. Overexpression of *HELLS* was reported in several types of cancers, e.g., nasopharyngeal carcinoma, hepatocellular carcinoma, colorectal cancer and pancreatic cancer [49,51,52,53]. In pancreatic cancer patients, the expression of *HELLS* is correlated with clinical stage and the prediction of short survival. Moreover, *HELLS* knockdown leads to cell cycle arrest and enhanced cisplatin sensitivity of pancreatic cancer cells [51]. Our present study of LUSQ cells confirmed that depletion of *HELLS* attenuates cancer cell aggressive phenotypes. Consequently, aberrant HELLS expression is closely linked to the malignant progression of several cancers, suggesting that HELLS is a candidate therapeutic target for these cancers.

In this study, a total of 49 putative target was identified as *miR-150-3p* regulation in LUSQ cells. Large-scale cohort studies of these genes may reveal new diagnostic markers for LUSQ. Furthermore, it is also an important issue to investigate the relationship between genomic mutations or treatment response in patients with LUSQ and the expression of these genes. Further analysis of miRNA-controlled molecular networks will lead to the search for diagnostic markers and therapeutic targets for this disease.

## 5. Conclusions

Analysis of the TCGA database showed that *miR-150-3p* expression was significantly downregulated in LUSQ clinical specimens. Ectopic expression assays demonstrated that *miR-150-3p* inhibited the aggressiveness of LUSQ cells, suggesting that *miR-150-3p* acts as a tumor suppressor. Interestingly, *miR-150-3p* was found to controlled cell cycle-related genes. The expression of *HELLS*, among the various cell cycle regulators, was demonstrated to be directly controlled by *miR-150-3p*, and its aberrant expression enhanced the malignant phenotypes of LUSQ cells. Results here and from our previous studies demonstrate that both strands of pre-*miR-150* (the *miR-150-5p* guide strand and *miR-150-3p* passenger strand) have extremely important tumor suppressor functions in LUSQ cells. Involvement of the passenger strand of miRNA in LUSQ molecular pathogenesis is a new concept in cancer research.

## Figures and Tables

**Table 1:** Candidate target genes regulated by miR-150-3p in LUSQ cells.

Entrez Gene ID	Gene Symbol	Gene Name	Total Sites	GSE19188 Log_2_ FC	EBC-1 *miR-150-3p* Transfectant Log_2_ FC	TCGA OncoLnc *p*-Value
1058	*CENPA*	centromere protein A	1	3.49	−1.56	0.097
83540	*NUF2*	NUF2 component of NDC80 kinetochore complex	2	3.44	−2.30	0.366
699	*BUB1*	BUB1 mitotic checkpoint serine/threonine kinase	1	3.21	−1.86	0.172
3070	*HELLS*	helicase, lymphoid specific	1	3.15	−1.75	0.002
4173	*MCM4*	minichromosome maintenance complex component 4	1	3.13	−2.07	0.315
150468	*CKAP2L*	cytoskeleton associated protein 2 like	2	3.03	−1.89	0.700
9837	*GINS1*	GINS complex subunit 1	1	2.99	−2.29	0.509
84951	*TNS4*	tensin 4	1	2.56	−1.05	0.908
157313	*CDCA2*	cell division cycle associated 2	1	2.39	−1.53	0.538
29028	*ATAD2*	ATPase family AAA domain containing 2	1	2.25	−1.96	0.329
898	*CCNE1*	cyclin E1	1	2.23	−1.51	0.255
57082	*CASC5*	cancer susceptibility candidate 5	1	2.23	−1.91	0.377
55771	*PRR11*	proline rich 11	2	2.20	−1.83	0.548
2146	*EZH2*	enhancer of zeste 2 polycomb repressive complex 2 subunit	1	2.06	−1.02	0.095
9134	*CCNE2*	cyclin E2	1	2.04	−2.73	0.175
221150	*SKA3*	spindle and kinetochore associated complex subunit 3	1	2.01	−2.53	0.616
84733	*CBX2*	chromobox 2	1	1.99	−1.16	0.422
1870	*E2F2*	E2F transcription factor 2	1	1.70	−2.87	0.584
8357	*HIST1H3H*	histone cluster 1 H3 family member h	1	1.70	−2.14	0.251
84798	*C19orf48*	chromosome 19 open reading frame 48	1	1.68	−1.35	0.159
8970	*HIST1H2BJ*	histone cluster 1 H2B family member j	1	1.66	−1.08	0.451
8914	*TIMELESS*	timeless circadian regulator	1	1.65	−2.16	0.433
79172	*CENPO*	centromere protein O	1	1.62	−1.48	0.259
26586	*CKAP2*	cytoskeleton associated protein 2	1	1.54	−1.20	0.454
94032	*CAMK2N2*	calcium/calmodulin dependent protein kinase II inhibitor 2	1	1.54	−2.37	0.994
55055	*ZWILCH*	zwilch kinetochore protein	1	1.53	−2.41	0.223
25886	*POC1A*	POC1 centriolar protein A	1	1.43	−2.10	0.084
79723	*SUV39H2*	suppressor of variegation 3–9 homolog 2	1	1.39	−1.58	0.261
81831	*NETO2*	neuropilin and tolloid like 2	2	1.38	−1.85	0.379
8358	*HIST1H3B*	histone cluster 1 H3 family member b	1	1.36	−2.24	0.127
5563	*PRKAA2*	protein kinase AMP-activated catalytic subunit alpha 2	1	1.32	−1.05	0.0382
54830	*NUP62CL*	nucleoporin 62 C-terminal like	1	1.32	−1.20	0.794
146956	*EME1*	essential meiotic structure-specific endonuclease 1	1	1.30	−1.69	0.234
11113	*CIT*	citron rho-interacting serine/threonine kinase	1	1.28	−2.49	0.553
201725	*C4orf46*	chromosome 4 open reading frame 46	1	1.28	−2.39	0.249
126433	*FBXO27*	F-box protein 27	1	1.27	−2.29	0.950
5738	*PTGFRN*	prostaglandin F2 receptor inhibitor	2	1.24	−2.51	0.645
22824	*HSPA4L*	heat shock protein family A (Hsp70) member 4 like	1	1.24	−1.02	0.889
140707	*BRI3BP*	BRI3 binding protein	1	1.23	−1.87	0.829
80179	*MYO19*	myosin XIX	1	1.19	−1.50	0.333
2177	*FANCD2*	FA complementation group D2	1	1.15	−1.54	0.403
9802	*DAZAP2*	DAZ associated protein 2	2	1.14	−1.78	0.479
79071	*ELOVL6*	ELOVL fatty acid elongase 6	1	1.13	−1.11	0.693
374655	*ZNF710*	zinc finger protein 710	1	1.11	−1.20	0.0206
145282	*MIPOL1*	mirror-image polydactyly 1	1	1.11	−1.03	0.254
10849	*CD3EAP*	CD3e molecule associated protein	1	1.10	−1.28	0.267
150223	*YDJC*	YdjC chitooligosaccharide deacetylase homolog	1	1.09	−1.49	0.352
80010	*RMI1*	RecQ mediated genome instability 1	1	1.01	−1.26	0.621
26355	*FAM162A*	family with sequence similarity 162 member A	1	1.01	−1.87	0.0475

FC: fold change; TCGA: The Cancer Genome Atlas.

**Table 2:** Significantly enriched annotations of target genes regulated by *miR-150-3p*.

Description	Annotation	Number of Genes	*p*-Value	Genes
cell cycle	GO:0007049	17	9.64 × 10^−12^	*CENPA, CIT, CCNE1, CCNE2, TIMELESS, BUB1, MCM4, HELLS, SKA3, CDCA2, FANCD2, NUF2, E2F2, SUV39H2, CASC5, ZWILCH, CKAP2*
cell division	GO:0051301	12	1.36 × 10^−8^	*CENPA, CIT, CCNE1, CCNE2, TIMELESS, BUB1, HELLS, SKA3, CDCA2, NUF2, CASC5, ZWILCH*
chromosome segregation	GO:0007059	5	0.000241047	*BUB1, SKA3, CDCA2, NUF2, CASC5*
chromatin organization	GO:0006325	7	0.00128717	*HIST1H3B, HIST1H3H, PRKAA2, EZH2, CBX2, SUV39H2, ATAD2*
DNA replication initiation	GO:0006270	3	0.00275067	*CCNE1, CCNE2, MCM4*
homologous chromosome pairing at meiosis	GO:0007129	3	0.0027733	*CCNE1, CCNE2, FANCD2*
CENP-A containing nucleosome assembly	GO:0034080	3	0.00566854	*CENPA, CENPO, CASC5*
negative regulation of gene expression, epigenetic	GO:0045814	3	0.00814561	*HIST1H3B, HIST1H3H, EZH2*
mitotic cytokinesis	GO:0000281	3	0.00864655	*CENPA, CIT, CKAP2*
regulation of gene silencing	GO:0060968	2	0.00873016	*HIST1H3B, HIST1H3H*

GO: Gene Ontology.

## Data Availability

Data is contained within the article or supplementary material.

## Supplementary Materials

The following are available online at https://www.mdpi.com/article/10.3390/biomedicines9121883/s1, Figure S1: Cell migration and invasion assays in LUSQ cells with ectopic expression of *miR-150-5p* or *miR-150-3p*. Figure S2: Cell proliferation was assessed with XTT assays after transfection of *miR-150-5p*, *-3p* or *5p + 3p*. Figure S3: Downregulation of HELLS protein expression by *miR-150-3p*. Figure S4: The nucleotide sequence of HELLS gene (NM_001289067.2). Figure S5: The sequence of the region containing the *miR-150-3p* binding site of each cell line. Figure S6: HELLS protein expression downregulated by si-*HELLS*. Figure S7: Cell migration and invasion assays in EBC-1 and SK-MES-1 after knockdown of HELLS by si-*HELLS.* Figure S8: Kaplan–Meier curves of 5-year overall survival due to difference in *HELLS* expression in LUSQ and LUAD patients from TCGA database. Table S1: Reagents used in this study. Table S2: Characteristics of the patients used for normal lung tissue near the tumor in Figure 2A. Table S3: Characteristics of the patients used for lung cancer tissue in Figure 2A. Table S4: Characteristics of the patients used for immunostaining.

## References

[1] Fitzmaurice C., Allen C., Barber R.M., Barregard L., Bhutta Z.A., Brenner H., Dicker D.J., Chimed-Orchir O., Dandona R., Dandona L. (2017). Global, regional, and national cancer incidence, mortality, years of life lost, years lived with disability, and disability-adjusted life-years for 32 cancer groups, 1990 to 2015: A systematic analysis for the global burden of disease study. JAMA Oncol..

[2] Bray F., Ferlay J., Soerjomataram I., Siegel R.L., Torre L.A., Jemal A. (2018). Global cancer statistics 2018: GLOBOCAN estimates of incidence and mortality worldwide for 36 cancers in 185 countries. CA Cancer J. Clin..

[3] Travis W.D., Brambilla E., Nicholson A.G., Yatabe Y., Austin J.H.M., Beasley M.B., Chirieac L.R., Dacic S., Duhig E., Flieder D.B. (2015). The 2015 World Health Organization classification of lung tumors: Impact of genetic, clinical and radiologic advances since the 2004 classification. J. Thorac. Oncol..

[4] Hida T., Nokihara H., Kondo M., Kim Y.H., Azuma K., Seto T., Takiguchi Y., Nishio M., Yoshioka H., Imamura F. (2017). Alectinib versus crizotinib in patients with ALK-positive non-small-cell lung cancer (J-ALEX): An open-label, randomised phase 3 trial. Lancet.

[5] Ramalingam S.S., Vansteenkiste J., Planchard D., Cho B.C., Gray J.E., Ohe Y., Zhou C., Reungwetwattana T., Cheng Y., Chewaskulyong B. (2020). Overall survival with osimertinib in untreated, EGFR-mutated advanced NSCLC. N. Engl. J. Med..

[6] Paik P.K., Felip E., Veillon R., Sakai H., Cortot A.B., Garassino M.C., Mazieres J., Viteri S., Senellart H., Van Meerbeeck J. (2020). Tepotinib in non-small-cell lung cancer with MET exon 14 skipping mutations. N. Engl. J. Med..

[7] Drilon A., Oxnard G.R., Tan D.S.W., Loong H.H.F., Johnson M., Gainor J., McCoach C.E., Gautschi O., Besse B., Cho B.C. (2020). Efficacy of selpercatinib in RET fusion-positive non-small-cell lung cancer. N. Engl. J. Med..

[8] Planchard D., Smit E.F., Groen H.J.M., Mazieres J., Besse B., Helland Å., Giannone V., D’Amelio A.M., Zhang P., Mookerjee B. (2017). Dabrafenib plus trametinib in patients with previously untreated BRAF(V600E)-mutant metastatic non-small-cell lung cancer: An open-label, phase 2 trial. Lancet Oncol..

[9] Drilon A., Siena S., Dziadziuszko R., Barlesi F., Krebs M.G., Shaw A.T., de Braud F., Rolfo C., Ahn M.J., Wolf J. (2020). Entrectinib in ROS1 fusion-positive non-small-cell lung cancer: Integrated analysis of three phase 1-2 trials. Lancet Oncol..

[10] Bartel D.P. (2009). MicroRNAs: Target recognition and regulatory functions. Cell.

[11] Bartel D.P. (2004). MicroRNAs: Genomics, biogenesis, mechanism, and function. Cell.

[12] Catalanotto C., Cogoni C., Zardo G. (2016). MicroRNA in control of gene expression: An overview of nuclear functions. Int. J. Mol. Sci..

[13] Lin S., Gregory R.I. (2015). MicroRNA biogenesis pathways in cancer. Nat. Rev. Cancer..

[14] Baer C., Claus R., Plass C. (2013). Genome-wide epigenetic regulation of miRNAs in cancer. Cancer Res..

[15] Di Leva G., Garofalo M., Croce C.M. (2014). MicroRNAs in cancer. Ann. Rev. Pathol..

[16] Gulyaeva L.F., Kushlinskiy N.E. (2016). Regulatory mechanisms of microRNA expression. J. Transl. Med..

[17] Ramassone A., Pagotto S., Veronese A., Visone R. (2018). Epigenetics and MicroRNAs in Cancer. Int. J. Mol. Sci..

[18] Ma L. (2016). MicroRNA and metastasis. Adv. Cancer Res..

[19] Bayraktar R., Van Roosbroeck K. (2018). miR-155 in cancer drug resistance and as target for miRNA-based therapeutics. Cancer Metastasis Rev..

[20] Desantis V., Saltarella I., Lamanuzzi A., Melaccio A., Solimando A.G., Mariggiò M.A., Racanelli V., Paradiso A., Vacca A., Frassanito M.A. (2020). MicroRNAs-based nano-strategies as new therapeutic approach in multiple myeloma to overcome disease progression and drug resistance. Int. J. Mol. Sci..

[21] Zhang S., Cheng Z., Wang Y., Han T. (2021). The risks of miRNA therapeutics: In a drug target perspective. Drug Des. Devel. Ther..

[22] Matranga C., Tomari Y., Shin C., Bartel D.P., Zamore P.D. (2005). Passenger-strand cleavage facilitates assembly of siRNA into Ago2-containing RNAi enzyme complexes. Cell.

[23] Koshizuka K., Nohata N., Hanazawa T., Kikkawa N., Arai T., Okato A., Fukumoto I., Katada K., Okamoto Y., Seki N. (2017). Deep sequencing-based microRNA expression signatures in head and neck squamous cell carcinoma: Dual strands of pre-miR-150 as antitumor miRNAs. Oncotarget.

[24] Yonemori K., Seki N., Idichi T., Kurahara H., Osako Y., Koshizuka K., Arai T., Okato A., Kita Y., Arigami T. (2017). The microRNA expression signature of pancreatic ductal adenocarcinoma by RNA sequencing: Anti-tumour functions of the microRNA-216 cluster. Oncotarget.

[25] Toda H., Kurozumi S., Kijima Y., Idichi T., Shinden Y., Yamada Y., Arai T., Maemura K., Fujii T., Horiguchi J. (2018). Molecular pathogenesis of triple-negative breast cancer based on microRNA expression signatures: Antitumor miR-204-5p targets AP1S3. J. Hum. Genet..

[26] Wada M., Goto Y., Tanaka T., Okada R., Moriya S., Idichi T., Noda M., Sasaki K., Kita Y., Kurahara H. (2020). RNA sequencing-based microRNA expression signature in esophageal squamous cell carcinoma: Oncogenic targets by antitumor miR-143-5p and miR-143-3p regulation. J. Hum. Genet..

[27] Sanada H., Seki N., Mizuno K., Misono S., Uchida A., Yamada Y., Moriya S., Kikkawa N., Machida K., Kumamoto T. (2019). Involvement of dual strands of miR-143 (miR-143-5p and miR-143-3p) and their target oncogenes in the molecular pathogenesis of lung adenocarcinoma. Int. J. Mol. Sci..

[28] Misono S., Seki N., Mizuno K., Yamada Y., Uchida A., Arai T., Kumamoto T., Sanada H., Suetsugu T., Inoue H. (2018). Dual strands of the miR-145 duplex (miR-145-5p and miR-145-3p) regulate oncogenes in lung adenocarcinoma pathogenesis. J. Hum. Genet..

[29] Toda H., Seki N., Kurozumi S., Shinden Y., Yamada Y., Nohata N., Moriya S., Idichi T., Maemura K., Fujii T. (2020). RNA-sequence-based microRNA expression signature in breast cancer: Tumor-suppressive miR-101-5p regulates molecular pathogenesis. Mol. Oncol..

[30] Koshizuka K., Hanazawa T., Kikkawa N., Arai T., Okato A., Kurozumi A., Kato M., Katada K., Okamoto Y., Seki N. (2017). Regulation of ITGA3 by the anti-tumor miR-199 family inhibits cancer cell migration and invasion in head and neck cancer. Cancer Sci..

[31] Suetsugu T., Koshizuka K., Seki N., Mizuno K., Okato A., Arai T., Misono S., Uchida A., Kumamoto T., Inoue H. (2018). Downregulation of matrix metalloproteinase 14 by the antitumor miRNA, miR-150-5p, inhibits the aggressiveness of lung squamous cell carcinoma cells. Int. J. Oncol..

[32] Misono S., Seki N., Mizuno K., Yamada Y., Uchida A., Sanada H., Moriya S., Kikkawa N., Kumamoto T., Suetsugu T. (2019). Molecular pathogenesis of gene regulation by the miR-150 duplex: miR-150-3p regulates TNS4 in lung adenocarcinoma. Cancers.

[33] Arai T., Okato A., Yamada Y., Sugawara S., Kurozumi A., Kojima S., Yamazaki K., Naya Y., Ichikawa T., Seki N. (2018). Regulation of NCAPG by miR-99a-3p (passenger strand) inhibits cancer cell aggressiveness and is involved in CRPC. Cancer Med..

[34] Lv Y., Yang H., Ma X., Wu G. (2019). Strand-specific miR-28-3p and miR-28-5p have differential effects on nasopharyngeal cancer cells proliferation, apoptosis, migration and invasion. Cancer Cell Int..

[35] Liu X., Ji Q., Zhang C., Liu X., Liu Y., Liu N., Sui H., Zhou L., Wang S., Li Q. (2017). miR-30a acts as a tumor suppressor by double-targeting COX-2 and BCL9 in H. pylori gastric cancer models. Sci. Rep..

[36] Okada R., Goto Y., Yamada Y., Kato M., Asai S., Moriya S., Ichikawa T., Seki N. (2020). Regulation of oncogenic targets by the tumor-suppressive miR-139 duplex (miR-139-5p and miR-139-3p) in renal cell carcinoma. Biomedicines.

[37] Koshizuka K., Hanazawa T., Kikkawa N., Katada K., Okato A., Arai T., Idichi T., Osako Y., Okamoto Y., Seki N. (2018). Antitumor miR-150-5p and miR-150-3p inhibit cancer cell aggressiveness by targeting SPOCK1 in head and neck squamous cell carcinoma. Auris Nasus Larynx.

[38] Osako Y., Seki N., Koshizuka K., Okato A., Idichi T., Arai T., Omoto I., Sasaki K., Uchikado Y., Kita Y. (2017). Regulation of SPOCK1 by dual strands of pre-miR-150 inhibit cancer cell migration and invasion in esophageal squamous cell carcinoma. J. Hum. Genet..

[39] Dai F.Q., Li C.R., Fan X.Q., Tan L., Wang R.T., Jin H. (2019). miR-150-5p inhibits non-small-cell lung cancer metastasis and recurrence by targeting HMGA2 and β-catenin signaling. Mol. Ther. Nucleic Acids..

[40] Bellon M., Lepelletier Y., Hermine O., Nicot C. (2009). Deregulation of microRNA involved in hematopoiesis and the immune response in HTLV-I adult T-cell leukemia. Blood.

[41] Wang P.Y., Li Y.J., Zhang S., Li Z.L., Yue Z., Xie N., Xie S.Y. (2010). Regulating A549 cells growth by ASO inhibiting miRNA expression. Mol. Cell. Biochem..

[42] Wu Z., Li W., Li J., Zhang Y., Zhang X., Xu Y., Hu Y., Li Q., Sun Q., Ma Z. (2020). Higher expression of miR-150-5p promotes tumorigenesis by suppressing LKB1 in non-small cell lung cancer. Pathol Res. Pract..

[43] Wu Q., Chen Y.F., Fu J., You Q.H., Wang S.M., Huang X., Feng X.J., Zhang S.H. (2014). Short hairpin RNA-mediated down-regulation of CENP-A attenuates the aggressive phenotype of lung adenocarcinoma cells. Cell. Oncol..

[44] Hu D.D., Chen H.L., Lou L.M., Zhang H., Yang G.L. (2020). SKA3 promotes lung adenocarcinoma metastasis through the EGFR-PI3K-Akt axis. Biosci Rep..

[45] Chen L., Yu J.H., Lu Z.H., Zhang W. (2015). E2F2 induction in related to cell proliferation and poor prognosis in non-small cell lung carcinoma. Int. J. Clin. Exp. Pathol..

[46] Zhang K., Zhang X., Cai Z., Zhou J., Cao R., Zhao Y., Chen Z., Wang D., Ruan W., Zhao Q. (2018). A novel class of microRNA-recognition elements that function only within open reading frames. Nat. Struct. Mol. Biol..

[47] Brümmer A., Hausser J. (2014). MicroRNA binding sites in the coding region of mRNAs: Extending the repertoire of post-transcriptional gene regulation. Bioessays.

[48] Lungu C., Muegge K., Jeltsch A., Jurkowska R.Z. (2015). An ATPase-deficient variant of the SNF2 family member HELLS shows altered dynamics at pericentromeric heterochromatin. J. Mol. Biol..

[49] Law C.T., Wei L., Tsang F.H., Chan C.Y., Xu I.M., Lai R.K., Ho D.W., Lee J.M., Wong C.C., Ng I.O. (2019). HELLS regulates chromatin remodeling and epigenetic silencing of multiple tumor suppressor genes in human hepatocellular carcinoma. Hepatology.

[50] Von Eyss B., Maaskola J., Memczak S., Möllmann K., Schuetz A., Loddenkemper C., Tanh M.D., Otto A., Muegge K., Heinemann U. (2012). The SNF2-like helicase HELLS mediates E2F3-dependent transcription and cellular transformation. EMBO J..

[51] Hou X., Yang L., Wang K., Zhou Y., Li Q., Kong F., Liu X., He J. (2021). HELLS, a chromatin remodeler is highly expressed in pancreatic cancer and downregulation of it impairs tumor growth and sensitizes to cisplatin by reexpressing the tumor suppressor TGFBR3. Cancer Med..

[52] Liu X., Hou X., Zhou Y., Li Q., Kong F., Yan S., Lei S., Xiong L., He J. (2019). Downregulation of the helicase lymphoid-specific (HELLS) gene impairs cell proliferation and induces cell cycle arrest in colorectal cancer cells. OncoTargets Ther..

[53] Waseem A., Ali M., Odell E.W., Fortune F., Teh M.T. (2010). Downstream targets of FOXM1: CEP55 and HELLS are cancer progression markers of head and neck squamous cell carcinoma. Oral Oncol..

