# Supplementary material for: Regulation of Oncogenic Targets by Tumor-Suppressive miR-150-3p in Lung Squamous Cell Carcinoma
Source: Biomedicines. 2021 Dec 11;9(12):1883. doi: 10.3390/biomedicines9121883 (PMC8698895; doi:10.3390/biomedicines9121883)
Supplement: Supplementary file 1 [file biomedicines-09-01883-s001.zip › biomedicines-1477163-supplementary.pdf]

biomedicines

MDPI

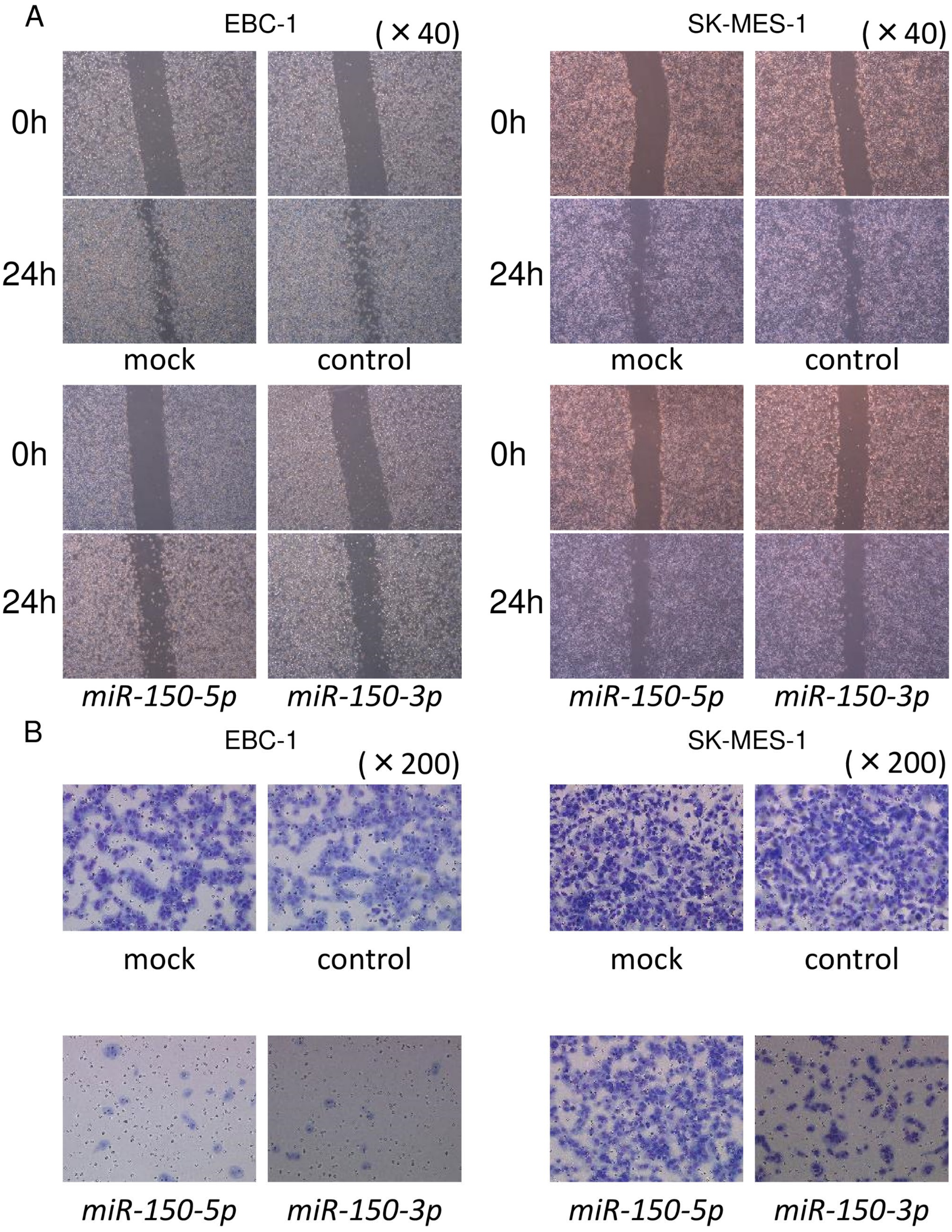


**Figure S1.** Cell migration and invasion assays in LUSQ cells with ectopic expression of *miR-150-5p* or *miR-150-3p*. (A) In wound healing assay, Phase-contrast micrographs of EBC-1 and SK-MES-1 cell lines transfected with *miR-150-5p* or *-3p* are shown. (B) Micrographs of LUSQ cells after transfection with *miR-150-5p* or *miR-150-3p* in Matrigel invasion assays are shown.

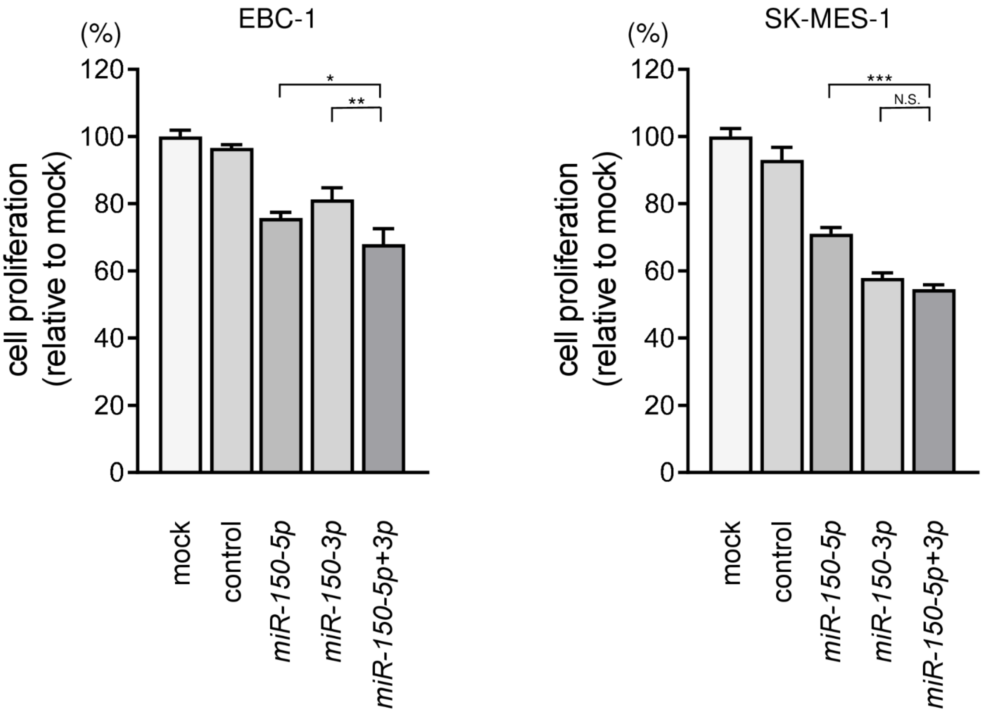


**Figure S2.** Cell proliferation was assessed with XTT assays after transfection of *miR-150-5p, -3p* or *5p + 3p*. Synergistic effects were not observed by combination of *miR-150-5p* and *miR-150-3p*. * $p < 0.05$, ** $p < 0.001$, *** $p < 0.01$, N.S.: not significant.

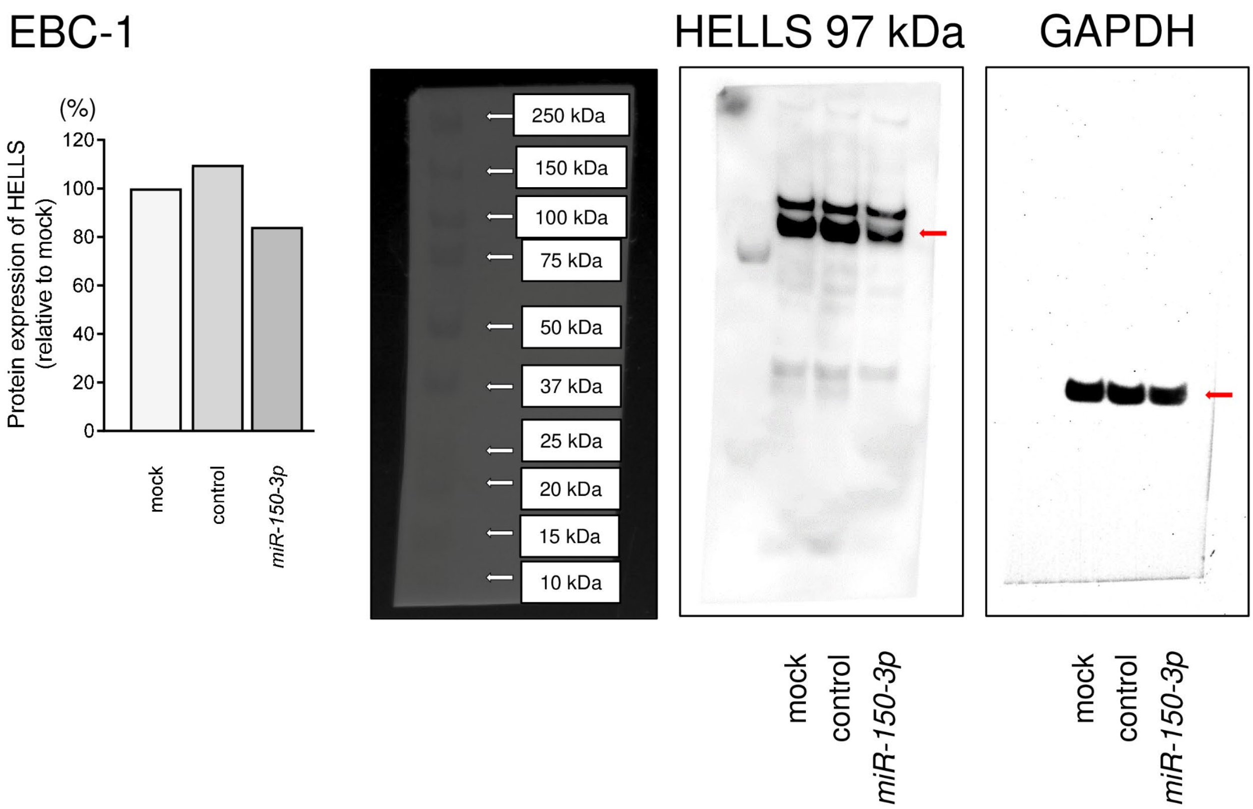


**Figure S3.** Downregulation of HELLS protein expression by *miR-150-3p*. Western blotting was performed to assay expression of HELLS protein. Full images of western blotting are shown. The western blotting results were quantified by ImageJ.

Homo sapiens helicase, lymphoid specific (HELLS), transcript variant 2, mRNA
NCBI Reference Sequence: NM_001289067.2
CDS 135--2789

1 gaaggagaag cgcgcttttt tccctggcgg gggatttggc tagaaggctg ggccggcagc
61 ggttgtgagg agttagctcg cggcattgca ggctctgaga ggaggggacc cggttcccgg
121 gtgagtgtcc aggcatgcca gcggaacggc ccgcgggcag cggcggctcg gaggctccag
181 caatggttga acaactggac actgctgtga ttaccccggc catgctagaa gaggaagaac
241 agcttgaagc tgctggacta gagagagagc ggaagatgct ggaaaaggct cgcatgtctt
301 gggatagaga gtcgacagaa attcggtacc gtagacttca acatttgctt gaaaaaagca
361 atatatactc caaattttta ttgacgaaaa tggaacagca acaattagag gaacagaaga
421 agaaagaaaa attggagaga aaaaaggagt ctttaaaagt taaaaagggt aaaaattcaa
481 ttgatgcaag tgaagagaag ccagttatga ggaaaaaaag aggaagagaa gatgaatcat
541 acaatatttc agaggtcatg tcaaaagagg aaattttgtc tgtggctaaa aaaaataaaa
601 aggagaatga ggatgaaaac tcctcctcta ctaatctctg tgtggaagat cttcagaaaa
661 ataaagattc gaatagtata attaaagata gattgtctga aacggttagg cagaatacta
721 aattcttttt tgacccagtc cggaagtgta atggtcagcc agtacctttt caacaaccaa
781 agcacttcac tggaggagtg atgcgatg**gt** **acca**agtaga aggcatggaa tggcttagga
841 tgctttggga aaatggaatt aatggcattt tagcagatga aatgggattg ggtaagacag
901 ttcagtgcat tgctactatt gcattgatga ttcagagagg agtaccagga ccttttcttg
961 tctgtggccc tttgtctaca cttcctaact ggatggctga attcaaaaga tttacaccag
1021 atatccctac aatgttatat catggaaccc aggaggaacg tcaaaaattg gtaagaaata
1081 tttacaaacg gaaagggact ttgcagattc atcctgtggt aatcacgtca tttgaaatag
1141 ccatgagaga ccgaaatgcg ttacagcatt gctattggaa atacttaata gtagatgaag
1201 gacacaggat taagaatatg aagtgccgtc taatcaggga gttaaaacga ttcaatgctg
1261 ataacaaact tcttttgact ggtactccct tgcaaaacaa tttatcagaa ctttggtcat
1321 tgctaaactt tttgttgcca gatgtatttg atgacttgaa aagctttgag tcttggtttg
1381 acatcactag tctttctgaa actgctgaag atattattgc taaagaaaga gaacagaatg
1441 tattgcatat gctgcaccag gaaagaattc ctagaatgta tgtgtcagag tttacagatg
1501 agggcttctt tgaaggcaaa ttgccctcca gaaaagattt atatgatata cgtccatatg
1561 aaaatactta tttactcaag cttcatacca attttgagat tttaacacct ttcttattga
1621 gaagactgaa gtctgatgtt gctcttgaag ttcctcctaa acgagaagta gtcgtttatg
1681 ctccactttc aaagaagcag gagatctttt atacagccat tgtgaaccgt acaattgcaa
1741 acatgtttgg atccagtgag aaagaaacaa ttgagttaag tcctactggt cgaccaaaac
1801 gacgaactag aaaatcaata aattacagca aaatagatga tttccctaat gaattggaaa
1861 aactgatcag tcaaatacag ccagaggtgg accgagaaag agctgttgtg gaagtgaata
1921 tccctgtaga atctgaagtt aatctgaagc tgcagaatat aatgatgcta cttcgtaaat
1981 gttgtaatca tccatatttg attgaatatc ctatagaccc tgttacacaa gaatttaaga
2041 tcgatgaaga attggtaaca aattctggga agttcttgat tttggatcga atgctgccag
2101 aactaaaaaa aagaggtcac aaggtgctgc tttttcaca aatgacaagc atgttggaca
2161 ttttgatgga ttactgccat ctcagagatt tcaacttcag caggcttgat gggtccatgt
2221 cttactcaga gagagaaaaa aacatgcaca gcttcaacac ggatccagag gtgtttatct
2281 tcttagtgag tacacgagct ggtggcctgg gcattaatct gactgcagca gatacagtta
2341 tcatttatga tagtgattgg aacccccagt cggatcttca ggcccaggat agatgtcata
2401 gaattggtca gacaaagcca gttgttgttt atcgccttgt tacagcaaat actatcgatc
2461 agaaaattgt ggaaagagca gctgctaaaa ggaaactgga aaagttgatc atccataaaa
2521 atcatttcaa aggtggtcag tctggattaa atctgtctaa gaatttctta gatcctaagg
2581 aattaatgga attattaaaa tctagagatt atgaaaggga aataaaagga tcaagagaga
2641 aggtcattag tgataaagat ctagagttgt tgttagatcg aagtgatctt attgatcaaa
2701 tgaatgcttc aggaccaatt aaagagaaga tggggatatt caagatatta gaaaattctg
2761 aagattccag tcctgaatgt ttgtttttaaa gtggagctca agaatagctt ttaaaagttc
2821 ttatttacat ctagtgattt ccctgtattg ggtttgaaat actgattgtc cacttcacct
2881 tttttattat atcagttgac atgtaactag taccatgcgt acttaaatag atggtaattt
2941 tctgagcctt accaagaaca aagaagtatc catattaagt ttagattttc agttaatttt
3001 tgagactgag tagtattctt ggatacaggc tgatgtgtac ttaaccactt ccagatttat
3061 acagtcttcc tgtggaagtt tagtaaatgt ctttttccct cctttcttct agtaatgcag
3121 ttcatgggct ttaggtactt cagttatgaa gtaggctttt catggggaga gattgggatt
3181 atgctctctg ttgtttaaga aactgtttga ttttagagtc tatttctatg agatagttta
3241 ccaaataaat gttccttata agatga

**Figure S4.** The nucleotide sequence of *HELLS* gene (NM_001289067.2). The first methionine (atg) and stop codon (taa) are shown in red. The binding sequences of *miR-150-3p* (gtacca) is shown in bold red.

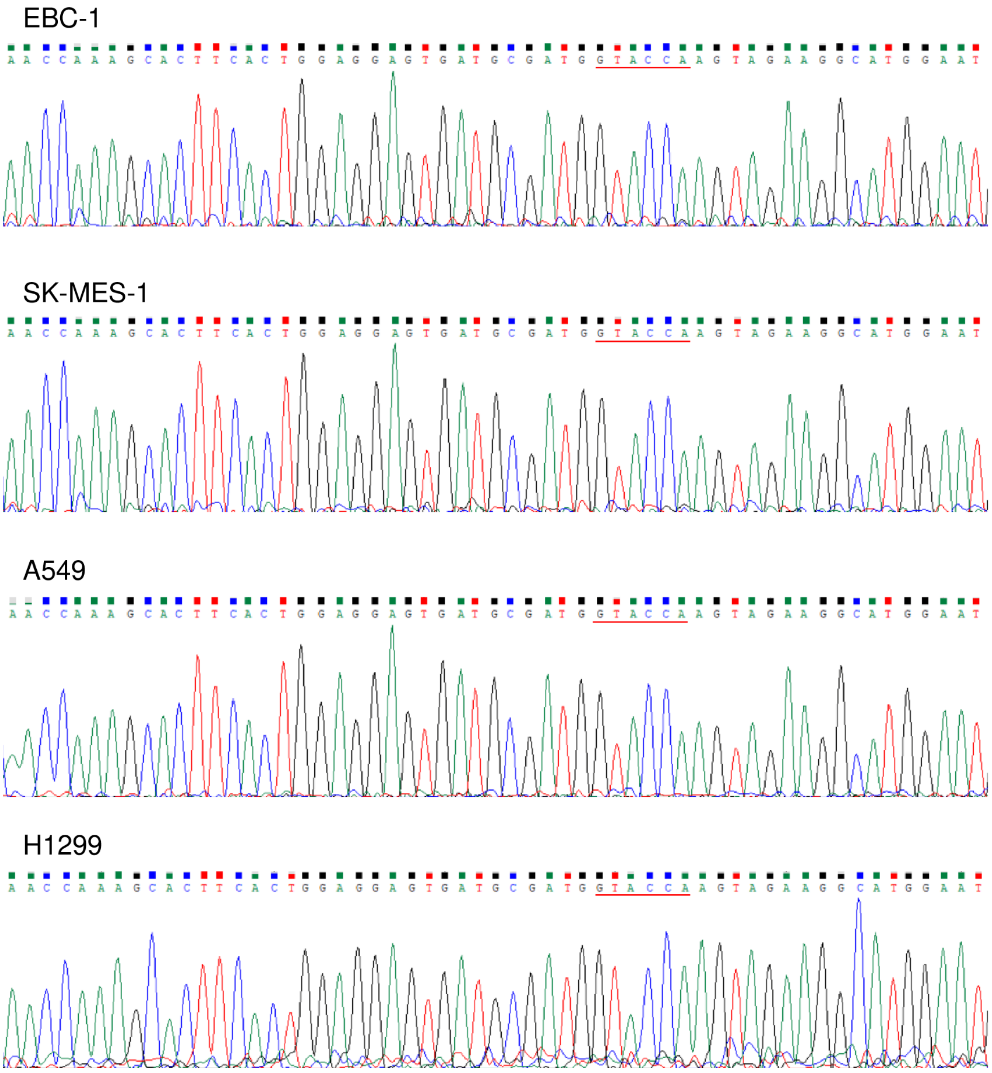


**Figure S5.** The sequence of the region containing the *miR-150-3p* binding site of each cell line. In each cell, a *miR-150-3p* binding sequence (gtacca) is present (red underline). This sequence was inserted into the luciferase reporter assay vector.

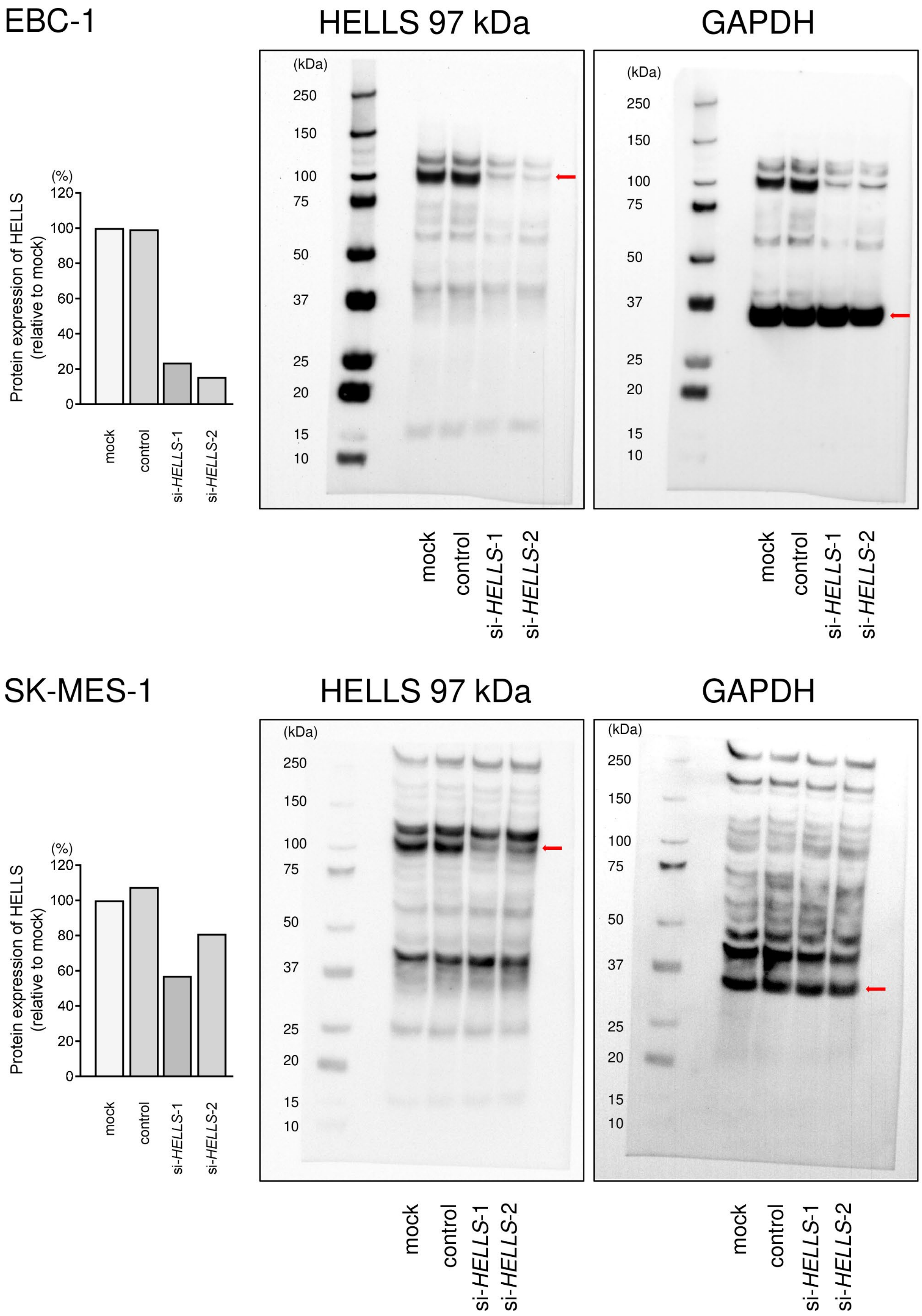


**Figure S6.** HELLS protein expression downregulated by si-*HELLS*. HELLS protein expression was evaluated by western blot. Full western blot images of HELLS are shown. The western blotting results were quantified by ImageJ.

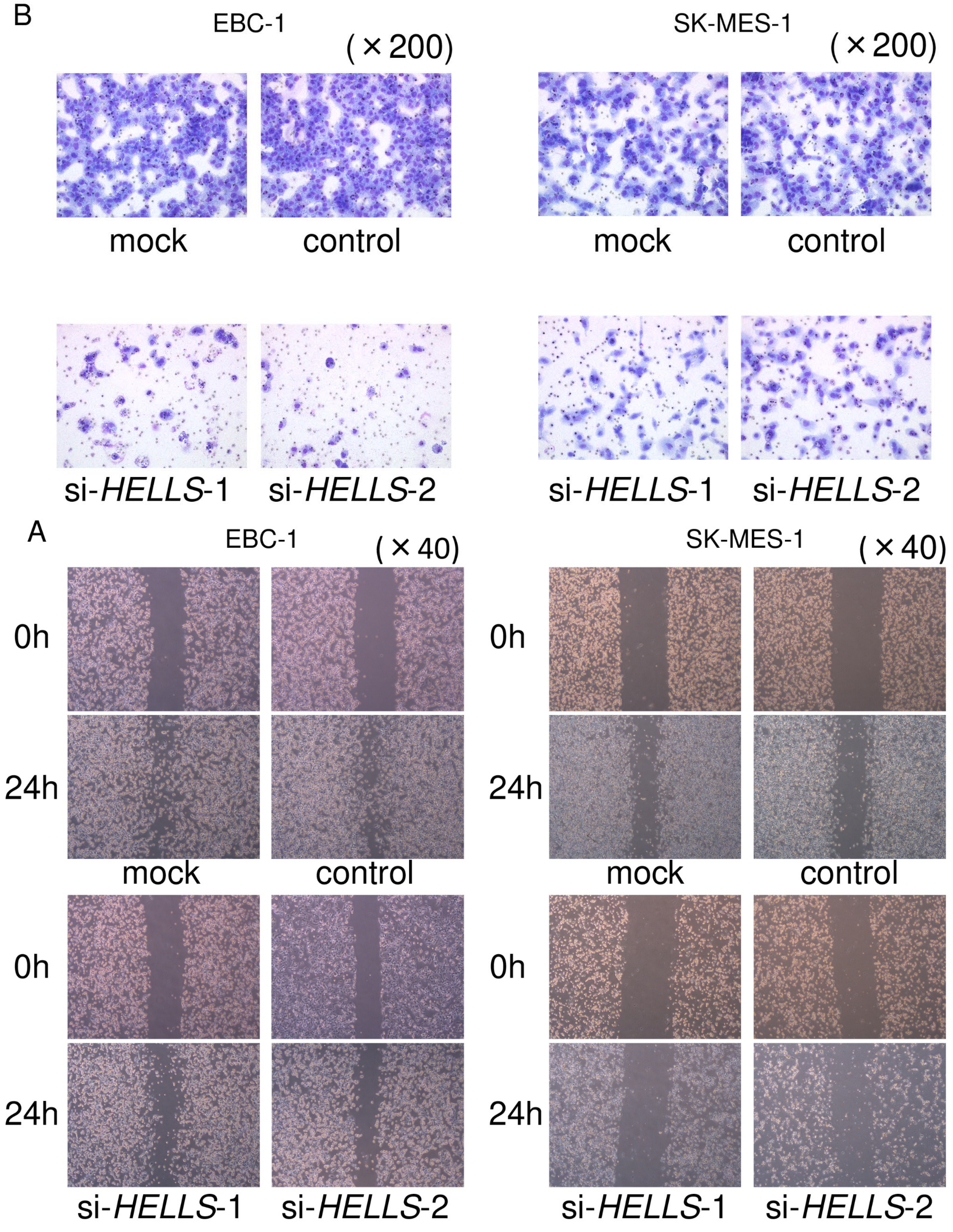


**Figure S7.** Cell migration and invasion assays in EBC-1 and SK-MES-1 after knockdown of HELLS by si-*HELLS*. (A) Phase-contrast micrographs of wound healing assay are shown after transfection with si-*HELLS* in EBC-1 and SK-MES-1. (B) Micrographs of Matrigel invasion assays for LUSQ cells transfected with si-*HELLS* are shown.

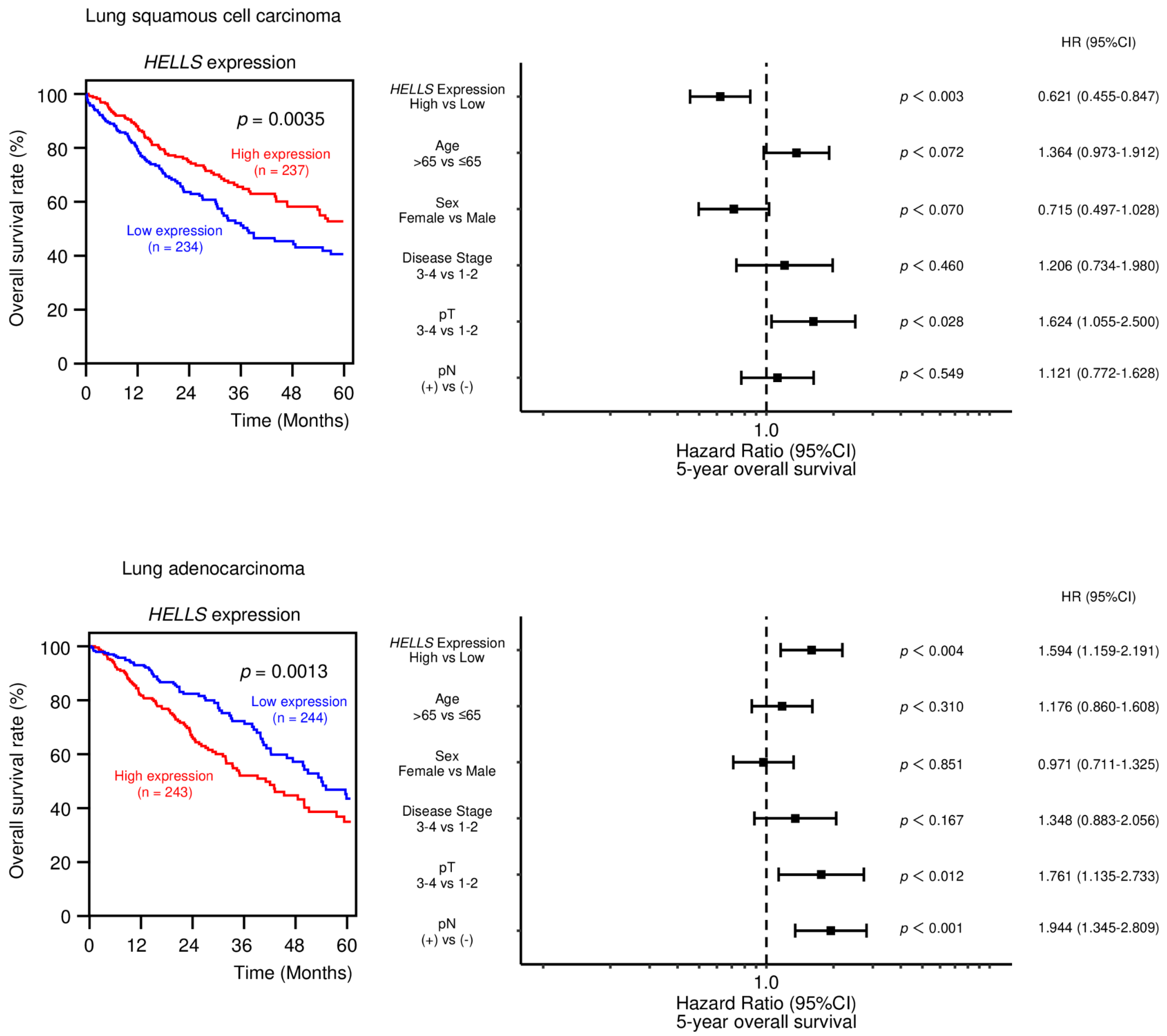


**Figure S8.** Kaplan–Meier curves of 5-year overall survival due to difference in *HELLS* expression in LUSQ and LUAD patients from TCGA database and forest plot presenting the results of a multivariate Cox regression analysis of the prognostic value of *HELLS* in TCGA-LUSQ and TCGA-LUAD. 5-year overall survival showed that low expression of *HELLS* was poor prognosis in LUSQ patients, but on the other hand high expression of *HELLS* was poor prognosis in LUAD. The expression level of *HELLS* was determined to be independent prognostic factors in terms of the 5-year overall survival rate after adjustments for age, sex, disease stage, pathological T and N stage ($p < 0.05$). HR: hazard ratio, CI: confidence interval.

**Table S1:** Reagents used in this study.

| Primer and probe | | Assay ID | Company |
|---|---|---|---|
| *miR-150-5p* | | 000473 | Applied Biosystem (Foster City, CA, USA) |
| *miR-150-3p* | | 002637 | Applied Biosystem |
| *RNU48* | | 001006 | Applied Biosystem |
| *HELLS* | | Hs00934778_m1 | Applied Biosystem |
| *GUSB* | | Hs00939627_m1 | Applied Biosystem |
| miRNA | Concentration | Catalog Number/Assay ID | Company |
| *miR-150-5p* | 10nM | AM17100 / PM10070 | Invitrogen (Carlsbad, CA, USA) |
| *miR-150-3p* | 10nM | AM17100 / PM12324 | Invitrogen |
| anti-miR Negative Control #1 | 10nM | AM17010 / AM1--100 | Invitrogen |
| siRNA | Concentration | Primer Name | Company |
| si-*HELLS* | 10nM | HSS104740, HSS104741, HSS179196 | Invitrogen (Carlsbad, CA, USA) |
| Antibody | Dilution | Catalog number | company |
| HELLS (SMARCA6) | WB 1:500 | ab3851 | Abcam (Cambridge, UK) |
| GAPDH | WB 1:10000 | MAB374 | EMD Millipore (Billerica, MA, USA) |
| HELLS | IHC 1:200 | HPA063242 | Sigma Aldrich (St. Louis, MO, USA) |

**Table S2:** Characteristics of the patients used for normal lung tissue near the tumor in Figure 2A.

| Age | Sex | T | N | M | Pathological stage | Type |
|---|---|---|---|---|---|---|
| 64 | M | 1a | 1 | 0 | IIA | LUSQ |
| 70 | M | 2a | 1 | 0 | IIA | LUSQ |
| 69 | M | 3 | 0 | 0 | IIB | LUSQ |
| 78 | M | 1a | 1 | 0 | IIA | LUSQ |
| 78 | M | 2b | 2a | 0 | IIIA | LUSQ |
| 79 | M | 3 | 1 | 0 | IIIA | LUSQ |
| 61 | M | 3 | 1 | 0 | IIIA | LUSQ |
| 75 | M | 1a | 0 | 0 | IA | LUSQ |
| 50 | M | 1a | 2 | 0 | IIIA | LUSQ |
| 73 | M | 2a | 0 | 0 | IB | LUSQ |
| 64 | M | 2a | 0 | 0 | IB | LUSQ |
| 88 | M | 3 | 0 | 0 | IIB | LUSQ |
| 68 | M | 3 | 0 | 0 | IIB | LUSQ |
| 76 | M | 1b | 0 | 0 | IA | LUSQ |
| 73 | M | 2a | 0 | 0 | IB | LUSQ |
| 76 | M | 2a | 0 | 0 | IB | LUSQ |
| 65 | M | 1b | 0 | 0 | IA | LUSQ |
| 69 | M | 1b | 0 | 0 | IA | LUSQ |
| 50 | M | 2a | 0 | 0 | IB | LUSQ |
| 65 | M | 2a | 0 | 0 | IB | LUSQ |
| 69 | M | 1a | 0 | 0 | IA | LUSQ |
| 65 | M | 2a | 0 | 0 | IB | LUSQ |

LUSQ: lung squamous cell carcinoma.

**Table S3:** Characteristics of the patients used for lung cancer tissue in Figure 2A.

| Age | Sex | T | N | M | Pathological stage | Type |
|---|---|---|---|---|---|---|
| 69 | M | 3 | 0 | 0 | IIB | LUSQ |
| 65 | M | 3 | 0 | 0 | IIB | LUSQ |
| 71 | M | 3 | 2 | 0 | IIIA | LUSQ |
| 78 | M | 2b | 2a | 0 | IIIA | LUSQ |
| 56 | M | 2a | 1 | 0 | IIA | LUSQ |
| 79 | M | 3 | 1 | 0 | IIIA | LUSQ |
| 61 | M | 3 | 1 | 0 | IIIA | LUSQ |
| 50 | M | 1a | 2 | 0 | IIIA | LUSQ |
| 74 | M | 3 | 0 | 0 | IIB | LUSQ |
| 73 | M | 2a | 0 | 0 | IB | LUSQ |
| 64 | M | 2a | 0 | 0 | IB | LUSQ |
| 88 | M | 3 | 0 | 0 | IIB | LUSQ |
| 68 | M | 3 | 0 | 0 | IIB | LUSQ |
| 65 | F | 1b | 2 | 0 | IIB | LUSQ |
| 72 | M | 2a | 1 | 0 | IIA | LUSQ |
| 76 | M | 1b | 0 | 0 | IA | LUSQ |
| 71 | M | 2a | 2 | 0 | IIIA | LUSQ |
| 73 | M | 2a | 0 | 0 | IB | LUSQ |
| 73 | M | 2a | 0 | 0 | IB | LUSQ |
| 71 | M | 2b | 2 | 0 | IIIA | LUSQ |
| 77 | M | 1a | 0 | 0 | IA | LUSQ |
| 73 | F | 3a | 0 | 0 | IIB | LUSQ |
| 82 | M | 2a | 0 | 0 | IB | LUSQ |
| 76 | M | 2a | 0 | 0 | IB | LUSQ |
| 65 | M | 1b | 0 | 0 | IA | LUSQ |
| 69 | M | 1b | 0 | 0 | IA | LUSQ |
| 50 | M | 2a | 0 | 0 | IB | LUSQ |
| 65 | M | 2a | 0 | 0 | IB | LUSQ |
| 69 | M | 1a | 0 | 0 | IA | LUSQ |
| 50 | M | 4 | 2 | 0 | IIIB | LUSQ |
| 65 | M | 2a | 0 | 0 | IB | LUSQ |

LUSQ: lung squamous cell carcinoma.

**Table S4:** Characteristics of the patients used for immunostaining.

| Patients | Age | Sex | T | N | M | Pathological stage | Type |
|---|---|---|---|---|---|---|---|
| A | 69 | M | 1 | 0 | 0 | IA | LUSQ |
| B | 48 | M | 2 | 0 | 0 | IB | LUSQ |
| C | 54 | M | 3 | 0 | 0 | IIB | LUSQ |
| D | 30 | M | - | - | - | - | Normal lung tissue |
| E | 24 | M | - | - | - | - | Normal lung tissue |
| F | 48 | M | - | - | - | - | Normal lung tissue |

LUSQ: lung squamous cell carcinoma.
